# Promoting Photocatalytic Carbon Dioxide Reduction by Tuning the Properties of Cocatalysts

**DOI:** 10.1002/chem.202203387

**Published:** 2023-01-13

**Authors:** Tokuhisa Kawawaki, Yuki Akinaga, Daichi Yazaki, Hinano Kameko, Daisuke Hirayama, Yuichi Negishi

**Affiliations:** ^1^ Department of Applied Chemistry Faculty of Science Tokyo University of Science Kagurazaka, Shinjuku-ku Tokyo 162-8601 Japan; ^2^ Research Institute for Science & Technology Tokyo University of Science Shinjuku-ku Tokyo 162-8601 Japan

**Keywords:** alloying, cocatalysts, CO_2_ reduction reaction, metal nanoparticles, morphology, photocatalysts

## Abstract

Suppressing the amount of carbon dioxide in the atmosphere is an essential measure toward addressing global warming. Specifically, the photocatalytic CO_2_ reduction reaction (CRR) is an effective strategy because it affords the conversion of CO_2_ into useful carbon feedstocks by using sunlight and water. However, the practical application of photocatalyst‐promoting CRR (CRR photocatalysts) requires significant improvement of their conversion efficiency. Accordingly, extensive research is being conducted toward improving semiconductor photocatalysts, as well as cocatalysts that are loaded as active sites on the photocatalysts. In this review, we summarize recent research and development trends in the improvement of cocatalysts, which have a significant impact on the catalytic activity and selectivity of photocatalytic CRR. We expect that the advanced knowledge provided on the improvement of cocatalysts for CRR in this review will serve as a general guideline to accelerate the development of highly efficient CRR photocatalysts.

## Graphical abstract

**Catalytic CRRiteria**: This review summarizes recent research and development trends in the improvement of cocatalysts, which have a significant impact on the catalytic activity and selectivity of photocatalytic carbon dioxide reduction reaction (CRR). It provides advanced knowledge on the improvement of cocatalysts for CRR, serving as a general guide to accelerate the development of highly efficient CRR photocatalysts.

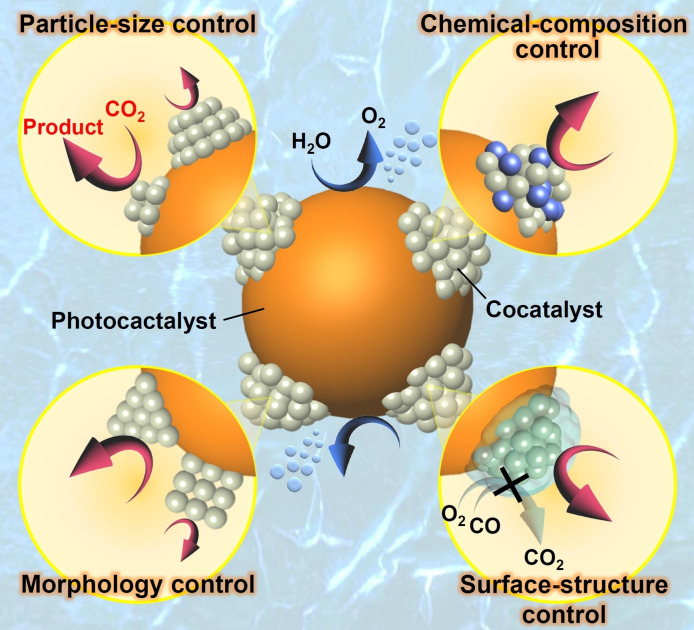

## 1 Introduction

### 1.1 Photocatalytic CO_2_ reduction

**Figure 1:**
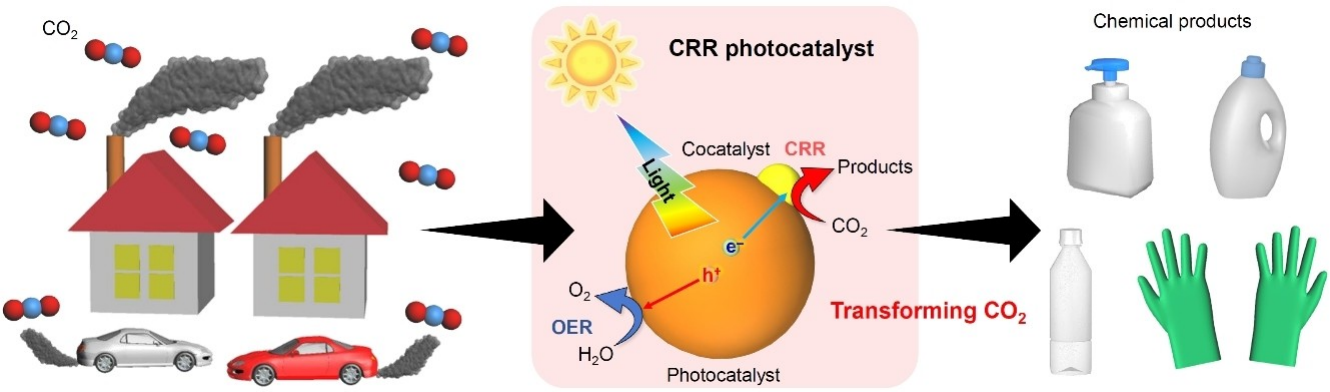
Schematic illustrations showing the transformation from CO_2_ in the atmosphere to useful carbon feedstocks.

Since the Industrial Revolution in the late 18th century, humankind has consumed large quantities of fossil fuels (coal, oil, and natural gas). The use of fossil fuels generates nitrides and sulfides, which are harmful to the ecosystem, cause air pollution, and increase greenhouse gas emissions (e. g., carbon dioxide), which ultimately lead to climate change. Therefore, reducing the use of fossil fuels and thereby suppressing CO_2_ emissions are pressing issues that need to be addressed to prevent global warming. In addition, given the current situation in which it is not possible to promptly and completely stop the use of fossil fuels and shift to a society that uses alternative greener fuels (e. g., hydrogen, ammonia) as its energy source, it is essential to establish means to reduce CO_2_ concentration in the atmosphere in parallel with reducing the use of fossil resources. As a strategy to reduce atmospheric CO_2_ concentration, a technology to capture and bury emitted CO_2_ underground has been established and is currently being applied. Alternatively, as a more efficient strategy, the emitted CO_2_ could be recycled (carbon cycle) through its conversion into carbon monoxide (CO) and organic compounds (synthesis gases and hydrocarbon compounds such as raw materials for chemicals, agrochemicals, and pharmaceuticals; Figure 1).

To reduce CO_2_ to useful compounds (CO_2_ reduction reaction (CRR)), photochemical reduction using sunlight and water (H_2_O) and electrochemical reduction using H_2_O are attracting attention as clean and sustainable technologies. However, between these two technologies, photochemical reduction, termed artificial photosynthesis, is more advantageous as it does not require complex device design or detailed components and it is therefore less expensive to implement. Consequently, photochemical methods are gaining more interest as an environmentally sustainable means of CRR. However, high conversion efficiencies from sunlight are yet to be achieved for CRR by artificial photosynthesis,^[1]^ for instance, through the fabrication of highly efficient photocatalysts/cocatalysts capable of high energy conversion efficiency from sunlight.

### 1.2 Cocatalysts for photocatalytic CRR

**Figure 2:**
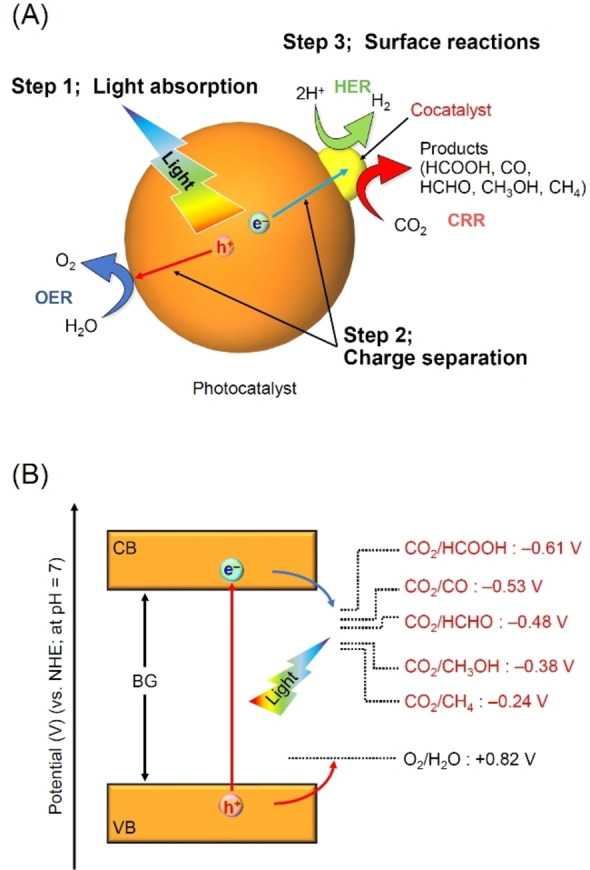
A) Schematic illustration of photocatalytic CRR: Step 1: light absorption, Step 2: charge separation, and Step 3: surface reactions. B) Principle of CRR using semiconductor photocatalysts.

In CRR, semiconductor photocatalysts absorb light energy and generate excited electrons (e^−^) with reduction power and excited holes (h^+^) with oxidizing power (Figure 2A). However, when these semiconductor photocatalysts are used for CRR, a high conversion efficiency cannot be obtained in most cases. This is largely due to: 1) deactivation of the carriers by exciton recombination and thermal relaxation in a relatively short period of time and 2) the small number of active sites on the photocatalyst surface that can serve as reaction sites. Thus, a cocatalyst (active site) consisting of metal and/or metal oxide particles is generally loaded on the photocatalyst (Figure 2A). By loading such cocatalysts, 1) the excited e^−^ or h^+^ generated within the photocatalyst are transferred to the cocatalyst, thereby extending the charge separation lifetime and 2) surface atoms and electronic states are created that are suitable for adsorption and desorption of the substrate, thereby improving the reactivity and selectivity of CRR. In addition, loading of the cocatalyst is effective in preventing self‐deactivation of the photocatalyst caused by its reducing or oxidizing power, thereby improving the durability of the photocatalyst.

Currently, the CRR process has the following limitations: 1) the reaction is difficult to proceed; 2) side reactions and reverse reactions are likely to occur; and 3) the selectivity of the reduction products is low. Limitation 1 is related to the fact that CO_2_ has a large negative standard Gibbs energy of evolution (−394.4 kJ mol^−1^) and is therefore a very stable molecule. As CO_2_ is more energetically stable than methanol (CH_3_OH; −166 kJ mol^−1^), methane (CH_4_; −51 kJ mol^−1^), or ethylene (C_2_H_4_; 68 kJ mol^−1^), the CRR process is an uphill reaction and reduction is thus difficult to proceed. Limitation 2 is related to the fact that the reduction potentials of CO_2_ and H_2_O are comparable to each other. This causes a competitive H_2_ evolution reaction (HER) to occur, in which H_2_O is reduced to H_2_, in parallel with CRR, making it difficult to only obtain the desired CO_2_‐reduced species with a high selectivity (Figure 2B). In addition, as oxygen (O_2_) and other substances produced in O_2_ evolution reaction (OER) are present in the system, a reverse reaction to CO_2_ can also occur upon reaction of these products and substances. Limitation 3 is related to the fact that there are various reducing species of CRR (e. g., CO, formic acid (HCOOH), formaldehyde (HCHO), acetic acid, CH_4_, and C_2_H_4_). Once such mixtures are obtained, their separation requires a great amount of energy. To overcome these challenges and realize artificial photosynthesis with high efficiency, high selectivity, and high durability, it is essential to improve the cocatalyst.^[2]^

### 1.3 Purpose and contents of this review

As described above, the improvement of cocatalysts plays an important role in the engineering of highly functional photocatalysts. Therefore, we have conducted extensive research on improving the cocatalysts on photocatalysts.^[3]^ Our work as well as the research conducted by other groups have provided much insights into the selection/fabrication of appropriate cocatalysts for water‐splitting photocatalysts.^[4]^ In contrast, the development of suitable cocatalysts for CRR is still in its infancy. To realize CRR with high efficiency, high selectivity, and high durability, a comprehensive review and summary of the findings and progress achieved to date is necessary. Therefore, in the present review, we summarize representative studies conducted to date on the design and engineering of cocatalysts in CRR using semiconductor photocatalysts.

In Section 2, the mechanism of CRR with semiconductor photocatalysts is described in detail, followed by a discussion on the characteristics and types of semiconductor photocatalysts used in CRR in Section 3. In Section 4, we describe examples of previous studies and findings on controlling the properties of cocatalysts, which is the subject of this review. Section 5 provides a summary of insights gained to date and an outlook on future prospects in this area.

The focus of the present review is on the control of cocatalyst particles in CRR using semiconductor photocatalysts. Therefore, readers interested in improving CRR photocatalysts using surface plasmon resonance,^[5]^ single‐atom metal loading,^[6]^ functional carbon,^[7]^ or metal complex loading^[8]^ are referred to those review articles.

## 2 Basis of Photocatalytic CRR

### 2.1 Mechanism

Photocatalytic CRR generally proceeds in H_2_O or in a vapor atmosphere. CRR proceeds according to the following three major steps (Figure 2A):[^2^, ^9^] Step 1) the semiconductor photocatalyst absorbs light, which leads to the evolution of excited e^−^ in the conduction band (CB) and excited h^+^ in the valence band (VB); Step 2) the excited e^−^ and h^+^ diffuse to the photocatalyst surface and, when a cocatalyst is present, further migrate to the cocatalyst; and Step 3) CRR [Eqs. (1)–(5)] or OER [Eq. (6)] proceeds on the cocatalyst or on the photocatalyst surface.

(1) $$\mathrm{CO}{}_{2}+2H{}^{+}+2e{}^{-}\to \mathrm{HCOOH}$$

(2) $$\mathrm{CO}{}_{2}+2H{}^{+}+2e{}^{-}\to \mathrm{CO}+H{}_{2}O$$

(3) $$\mathrm{CO}{}_{2}+4H{}^{+}+4e{}^{-}\to \mathrm{HCHO}+H{}_{2}O$$

(4) $$\mathrm{CO}{}_{2}+6H{}^{+}+6e{}^{-}\to \mathrm{CH}{}_{3}\mathrm{OH}+H{}_{2}O$$

(5) $$\mathrm{CO}{}_{2}+8H{}^{+}+8e{}^{-}\to \mathrm{CH}{}_{4}+2H{}_{2}O$$

(6) $$2H{}_{2}O+4\;h{}^{+}\to 4H{}^{+}+O{}_{2}$$

In a typical CRR, a proton (H^+^) is included in the reaction system [Eqs. (1)–(5)]. This is because of the high stability of CO_2_ that requires a large reducing power of −1.9 eV (vs. normal H_2_ electrode (NHE); at pH 7) for its conversion into an anion radical species. Such a large reduction power is difficult to generate with visible‐light‐driven semiconductor photocatalysts. In contrast, in multi‐electron reactions using H^+^ [Eqs. (1)–(5)], HCOOH, CO, HCHO, CH_3_OH, and CH_4_ are the reaction products. The reduction potential required for these reactions to proceed is much lower than that in one‐electron reactions (reactions via anion radical species). Therefore, most CRR processes that employ semiconductor photocatalysts are designed as multi‐electron reaction systems using H^+^.

### 2.2 Reaction system

**Figure 3:**
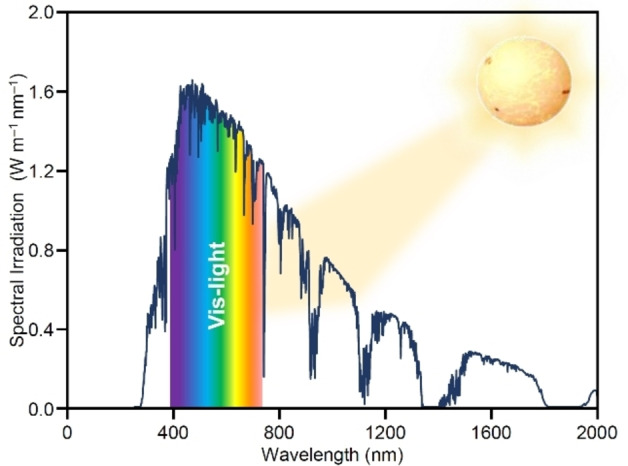
The sunlight spectrum. Visible light (∼400≤*λ*≤∼800 nm) accounts for about 43 % of sunlight.

**Figure 4:**
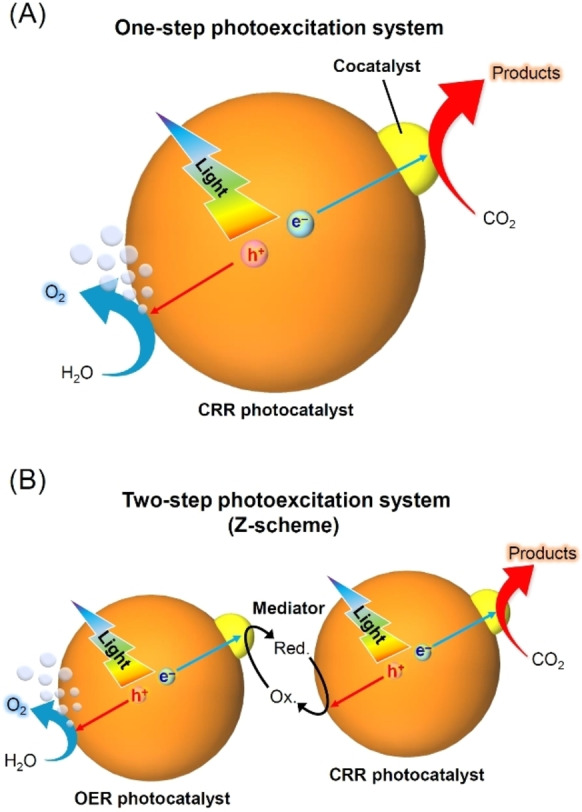
Schematic illustrations of photocatalytic reactions: A) one‐step photoexcitation system for CRR and B) Z‐scheme photoexcitation system for CRR. Red. and Ox. denote reductants and oxidants, respectively.

Theoretically, CRR proceeds when the CB minimum edge (CBM) of the semiconductor photocatalyst is more negative than the reduction potential of CO_2_. OER proceeds when the VB maximum edge (VBM) of the semiconductor photocatalyst is more positive than the oxidation potential of H_2_O (Figure 2B). Control of the CBM and VBM positions is relatively easy to achieve for semiconductor photocatalysts of sufficiently large band gaps (BGs; UV‐light‐driven semiconductor photocatalysts). However, as shown in Figure 3, visible light (∼400≤*λ*≤∼800 nm) accounts for about 43 % of sunlight. Therefore, it is indispensable to make effective use of sunlight using photocatalysts with small BGs, which can absorb visible light (namely, visible‐light‐driven semiconductor photocatalysts), for engineering semiconductor photocatalysts with high efficiencies. However, it is difficult to achieve appropriate CBM and VBM positions simultaneously in such semiconductor photocatalysts. Therefore, only a limited number of one‐step photocatalytic materials (Figure 4A) that can reduce CO_2_ under visible light have been reported.

In contrast, CRR can also proceed according to a two‐step reaction mechanism involving a semiconductor photocatalyst capable of instigating both CRR and OER. These reactions function by combining a photocatalyst, which causes CRR and OER, and a redox couple (mediator), which is responsible for the transfer of excitons (e^−^ and h^+^; Figure 4B). Such a system that mimics plant photosynthesis is called a Z‐scheme type. When using such Z‐scheme, there are significantly more types of photocatalysts available for study, and it is easier to use longer wavelength light with these photocatalysts than with the one‐step photocatalysts. However, there are some limitations such as the theoretically low conversion efficiency, blocking of light absorption, and photocatalyst inactivation due to side reactions resulting from the use of a mediator.

## 3 Photocatalysts Used for CRR

**Figure 5:**
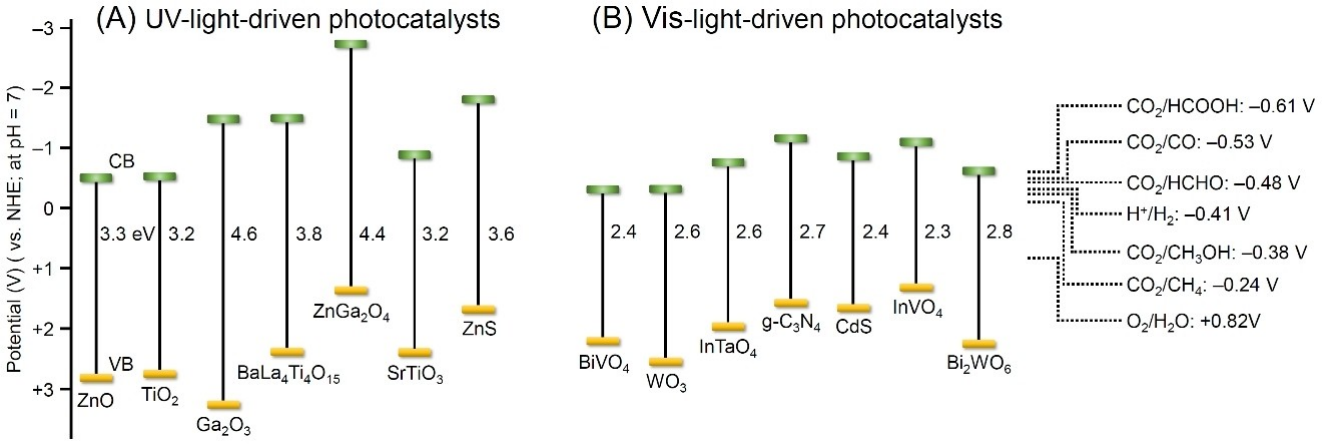
Band structures of A) UV‐ and B) visible‐light‐driven photocatalysts for CRR. The band position of the photocatalyst is calculated to be at pH 7.[^12^, ^15^]

Since Honda and colleagues reported using a semiconductor for CRR in 1979,^[10]^ various types of CRR photocatalysts have been subsequently developed. To date, the semiconductor photocatalysts include metal oxides,^[11]^ metal sulfides,^[12]^ metal‐free photocatalysts such as graphitic carbon nitride (g‐C_3_N_4_),^[13]^ and metal‐organic frameworks (MOFs).[^8a^, ^14^] These semiconductor photocatalysts have a band structure suitable for CRR (Figure 5).[^12^, ^15^]

Because metal and O_2_ form a strong bond, metal oxides are highly stable in air and H_2_O. Therefore, metal oxides are often used as photocatalytic materials for the fabrication of semiconductor photocatalysts. Among the range of metal oxides available, those containing d^0^ metal elements (transition metal ions such as titanium ion (Ti^4+^), zirconium ion (Zr^4+^), niobium ion (Nb^5+^), tantalum ion (Ta^5+^), vanadium ion (V^5+^), tungsten ion (W^6+^), and cerium ion (Ce^4+^)) and d^10^ metal elements (typical metal ions such as zinc ion (Zn^2+^), indium ion (In^3+^), gallium ion (Ga^3+^), germanium ion (Ge^4+^), tin ion (Sn^4+^), and antimony ion (Sb^5+^)) can function as CRR photocatalysts (e. g., TiO_2_, Ga_2_O_3_, WO_3_, and ZnO).[^15a^, ^15b^, ^15c^, ^16^] Photocatalysts such as BaLa_4_Ti_4_O_15_, SrTiO_3_, and ZnGa_2_O_4_, which contain several metal elements, are also used for CRR.^[17]^ Among them, metal oxides such as BiVO_4_, Bi_2_WO_6_, InTaO_4_, and InVO_4_ have a narrow BG and thereby absorb visible light.[^15d^, ^15g^, ^15j^, ^18^] Visible‐light‐driven photocatalysts can also be obtained by modifying the electronic structure of the UV‐light‐driven metal oxide photocatalysts. Representative approaches of achieving such modification are by: 1) hifting the energy position of the VBM to a negative potential by anion or metal cation substitution;^[19]^ 2) forming impurity levels in the BG by doping;^[20]^ and 3) narrowing the BG through the formation of a solid solution.^[21]^ Accordingly, several visible‐light‐driven metal‐oxide‐based photocatalysts have been developed and reported to date through the application of these modification processes.

Typical examples of metal sulfide semiconductor photocatalysts include CdS and ZnS.[^12^, ^22^] In contrast to the VB of metal oxides that is composed of O 2p orbitals, the VB of metal sulfides is composed of S 3p orbitals, which are located on the more negative side than the O 2p orbitals. Therefore, the BG of metal sulfides has a suitable width to absorb visible light, and CRR can be performed on metal sulfide photocatalysts under visible light irradiation. However, metal sulfides are readily oxidized by the h^+^ generated upon light irradiation, resulting in self‐decomposition and reduced durability of the photocatalyst. However, when the reaction is carried out in an aqueous solution containing sulfur species, self‐decomposition is suppressed, and a relatively high activity is achieved.

g‐C_3_N_4_ is an organic semiconductor photocatalyst that does not include a metal element.[^13^, ^15h^, ^23^] g‐C_3_N_4_ can be synthesized by a simple method of thermal polymerization[^15h^, ^24^] using N‐containing precursors (e. g., urea, melamine, and cyanamide). Moreover, these precursors are naturally abundant hence g‐C_3_N_4_ can be synthesized at a low cost. g‐C_3_N_4_ has a BG of 2.7–2.9 eV, which is suitable for visible light absorption, and the band structure is also suitable for CRR because the CBM is composed of C p_*z*_ orbitals and the VBM is composed of N p_*z*_ orbitals. Because of these desirable properties, g‐C_3_N_4_ has attracted great attention as a visible‐light‐driven photocatalytic material for CRR.

MOFs, which are composed of metal ions/clusters and organic linker molecules, have also been extensively studied as a photocatalytic material for CRR.[^8a^, ^14a^, ^14b^, ^25^] In MOFs, charge separation occurs efficiently because of their large specific surface area and high density of catalytic activity sites. These characteristics make MOFs attractive as a CRR photocatalyst.

## 4 Cocatalyst Used for Photocatalytic CRR

**Figure 6:**
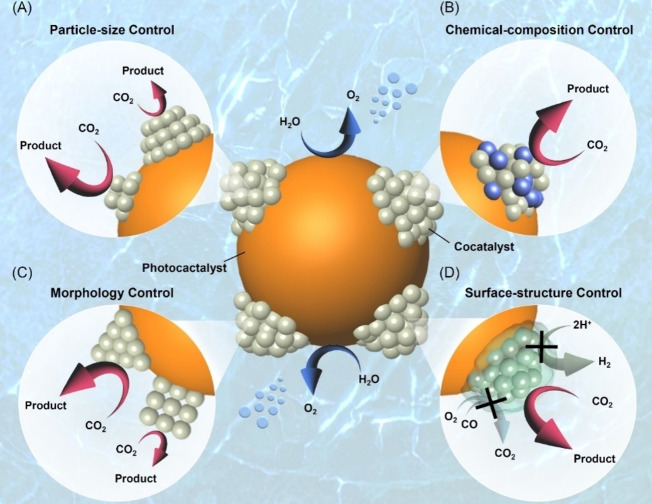
Schematic illustrations of photocatalyst functionalization by controlling the A) particle size (Section 4.1), B) chemical composition (Section 4.2), C) morphology (Section 4.3), and D) surface structure (Section 4.4) of the cocatalyst.

As described in Section 3, the use of semiconductor photocatalysts with a suitable band structure enables CRR to proceed. However, as discussed in Section 2, the use of semiconductor photocatalysts alone often results in, for instance, low catalytic activity and the simultaneous evolution of multiple by‐products even when the reduction reaction occurs. To overcome these limitations, metal nanoparticles (NPs) are often loaded on the surface of the semiconductor photocatalyst as cocatalysts. Previous studies have reported that metal NPs or metal oxide NPs consisting of platinum,^[26]^ palladium,[^26l^, ^26u^, ^26ab^, ^26af^, ^27^] gold,[^26l^, ^26aa^, ^26ab^, ^26ac^, ^26ai^, ^26aj^, ^27k^, ^28^] rhodium,[^26z^, ^26al^, ^29^] silver,[^26l^, ^26ac^, ^26ai^, ^26aj^, ^26am^, ^27k^, ^30^] ruthenium,[^26aj^, ^26ak^, ^28b^, ^31^] copper, –[^26aj^, ^27g^, ^27k^, ^32^] nickel,[^27h^, ^32n^, ^33^] molybdenum,^[34]^ titanium,[^32x^, ^35^] indium,^[36]^ or iridium,^[37]^ can function as CRR cocatalysts. In particular, Pt and PdNPs are used for the hydrogenation reactions because they are favorable for the adsorption and desorption of protons and thereby easy to produce CH_4_. Cu is used to form a C−C bond whose formation is important for producing C_2_ products, such as ethane (C_2_H_6_) and ethanol (C_2_H_5_OH). To improve CRR activity and selectivity, controlling the properties of the cocatalyst is essential. Four examples of cocatalyst property control are discussed subsequently: 1) particle size control (Section 4.1; Figure 6A);^[38]^ 2) chemical composition control (Section 4.2; Figure 6B);[^38c^, ^39^] 3) morphology control (Section 4.3; Figure 6C);[^39d^, ^40^] and 4) surface structure control (Section 4.4; Figure 6D).^[41]^ Types of cocatalyst and photocatalysts, CRR products, and other information in the literatures are summarized in Table 1.

**Table 1:** Cocatalysts, size of cocatalysts, photocatalysts, CRR products, light sources, selectivities, and references for the selected literature.

Section	Cocatalyst [wt%]	Size of cocatalyst [nm]	Photocatalysts	CRR products	Light sources	Selectivity [%]	Ref.
4.1 (particle size)	PtNPs (N/A)^[a]^	1.04±0.08	TiO_2_	CH_4_	400 W Xe lamp, at 250–388 nm	N/A^[a]^	[38a]
	PtNPs (1.6)	1.8	TiO_2_‐SiO_2_(HTSO)	CO, CH_4_	300 W Xe lamp, AM 1.5 filter^[b]^	39.1^[c]^	[38b]
4.2 (chemical composition)	Pd_7_Cu_1_ NPs (N/A)^[a]^	∼6	TiO_2_	CH_4_	300 W Xe lamp, >400 nm	95.9^[c]^	[39a]
	Pd_1_Cu_2_ NPs (1.69)	8.8	g‐C_3_N_4_	CH_4_	300 W Xe lamp, at 420–780 nm	96.5^[c]^	[39b]
	Pt_0.4_Cu_0.6_ NPs (N/A)^[a]^	N/A ^[a]^	TiO_2_	CH_4_	300 W Xe lamp, at 300–400 nm	100^[d]^	[39c]
4.3 (morphology)	tetra‐PdNPs (5.8)	4.9	g‐C_3_N_4_	CO, C_2_H_5_OH, CH_4_	300 W Xe lamp, >400 nm	78.1^[e]^	[40a]
	tetra‐PdNPs (0.39)	3.2	g‐C_3_N_4_	CH_4_, CH_3_OH	300 W Xe lamp	N/A^[a]^	[40b]
	concave cube‐PtCuNPs (8.5)	∼10.0	C_3_N_4_	CO, CH_4_	300 W Xe lamp, at 400–780 nm	90.6^[c]^	[39d]
4.4 (surface structure)	Cu_2_O/PtNPs (Cu: 1.70, Pt: 0.92)	3.1±0.5	TiO_2_	CO, CH_4_	200 W Xe lamp, at 320–780 nm	85.0^[f]^	[41a]
	Cr(OH)_3_ ⋅ *x*H_2_O/AgNPs (Cr: 1.00, Ag: 1.00)	N/A ^[a]^	Ga_2_O_3_	CO	400 W Hg lamp	83.8^[g]^	[41b]
	Cr(OH)_3_ ⋅ *x*H_2_O/AgNPs (Cr: 0.25, Ag: 0.25)	N/A ^[a]^	Ga_2_O_3_	CO	400 W Hg lamp	83.8^[g]^	[41c]

[a] N/A represent not applicable. [b] AM1.5G represent global standard solar spectrum (AM1.5G). [c] Selectivity in CH_4_ evolution is evaluated based on the required electrons using the following equation: Selectivity (%)=[8ν(CH_4_)]/[2ν(CO)+8ν(CH_4_)+2ν(H_2_)]×100, where ν(H_2_), ν(CO) and ν(CH_4_) stand for the evolution rates for H_2_, CO and CH_4_, respectively. [d] Selectivity in CH_4_ evolution is evaluated based on the required electrons using the following equation: Selectivity (%)=*n*(CH_4_)/[*n*(CO)+*n*(CH_4_)]×100, where *n*(CO) and *n*(CH_4_) stand for number of reacted electrons for CO and CH_4_, respectively. [e] Selectivity in carbon product evolution is evaluated based on the required electrons using the following equation: Selectivity (%)=[2ν(CO)+12ν(C_2_H_5_OH)+8ν(CH_4_)]/[2ν(CO)+12ν(C_2_H_5_OH)+8ν(CH_4_)+2ν(H_2_)]×100, where ν(H_2_), ν(CO), ν(CH_4_) and ν(C_2_H_5_OH) stand for the evolution rates for H_2_, CO, CH_4_, and C_2_H_5_OH, respectively. [f] Selectivity in carbon product evolution is evaluated based on the required electrons using the following equation: Selectivity (%)=[2*n*(CO)+8*n*(CH_4_)]/[2*n*(CO)+8*n*(CH_4_)+2*n*(H_2_)]×100, where *n*(H_2_), *n*(CO), and *n*(CH_4_) stand for the amounts (moles) of H_2_, CO, and CH_4_, respectively. [g] Selectivity in CO evolution is evaluated based on the required electrons using the following equation: Selectivity (%)=[2ν(CO)]/[2ν(CO)+2ν(H_2_)]×100, where ν(H_2_), and ν(CO) stand for the evolution rates for H_2_, and CO, respectively.

### 4.1 Particle size control

**Figure 7:**
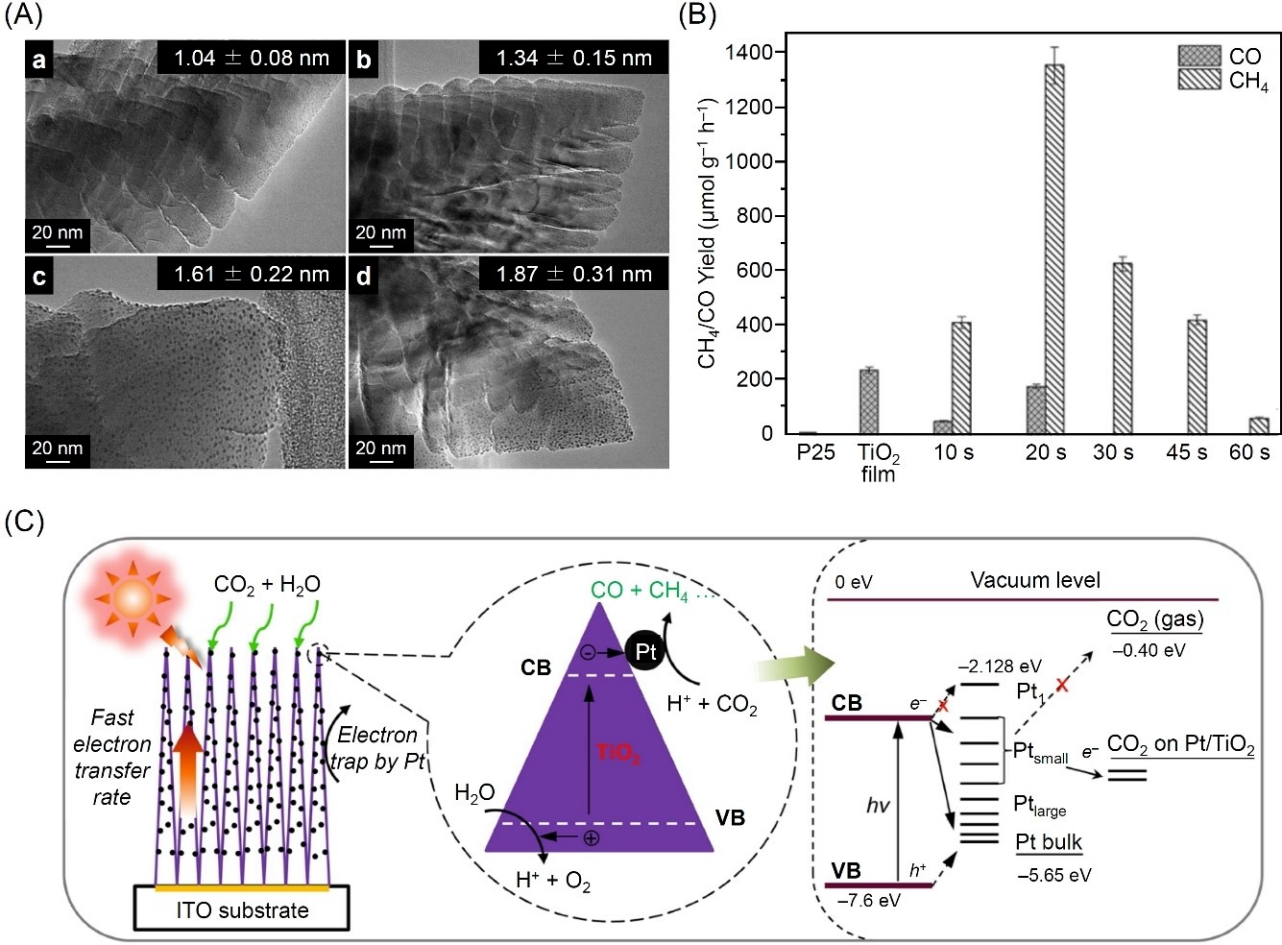
A) Transmission electron microscope (TEM) images of PtNPs/TiO_2_ prepared by using different Pt deposition times: 20 (a), 30 (b), 45 (c), and 60 s (d). B) CO and CH_4_ yields obtained during CRR on commercially available TiO_2_ powder (P25), pristine TiO_2_ columnar film (TiO_2_ film), and PtNPs/TiO_2_ films prepared by using different Pt deposition times (10, 20, 30, 45, and 60 s). C) Schematic diagram of CO_2_‐photoreduction mechanism with PtNPs/TiO_2_ nanostructured films. The magnified circle (center) shows that the photogenerated e^−^ can move rapidly within the highly oriented TiO_2_ single crystals and flow to the Pt deposits, where a redox reaction occurs to convert CO_2_ into CO and CH_4_. The energy levels of the PtNPs/TiO_2_‐CO_2_ system is also shown. Reproduced with permission from ref. [38a]. Copyright: 2012, American Chemical Society.

The size of the metal NPs that serve as cocatalysts has a significant effect on CRR activity and selectivity.^[38]^ Biswas and colleagues investigated the effect of PtNP cocatalysts of different particle sizes on CRR activity in 2012.^[38a]^ PtNPs of different particle sizes were formed on TiO_2_ (PtNPs/TiO_2_) by changing the deposition time of Pt atoms using a sputtering method (Figure 7A). When Pt was deposited for 20 s, small PtNPs (1.04±0.08 nm) were loaded on the photocatalyst. The resulting photocatalyst showed high activity (1361 μmol g^−1^ h^−1^), as assessed by the rate of CH_4_ evolution, with an estimated quantum yield of 2.41 % (Figure 7B). In comparison, when Pt was deposited for either a shorter time (e. g., 10 s) or a longer time (e. g., ≥30 s), smaller (0.63±0.06 nm) or larger (≥1.34±0.15 nm) PtNPs were loaded. These resulting photocatalysts showed lower activity for CH_4_ evolution than the photocatalyst loaded with 1.04±0.08 nm PtNPs (Figure 7B). The different CH_4_ evolution rates displayed by the PtNPs/TiO_2_ photocatalysts were attributed to changes in the Fermi level of the cocatalyst (Figure 7C). If the PtNPs are too small, electron transfer from the CB of TiO_2_ to PtNPs is unlikely to occur because the PtNPs have discrete energy bands. In contrast, if the PtNPs are too large, they can easily become the recombination center for e^−^ and h^+^ owing to their electronic structure, which is comparable to that of the bulk metal. These results show that to improve the catalytic activity of CRR, it is essential to obtain an appropriate electronic structure, which can be achieved by controlling the size of the cocatalyst particles.

**Figure 8:**
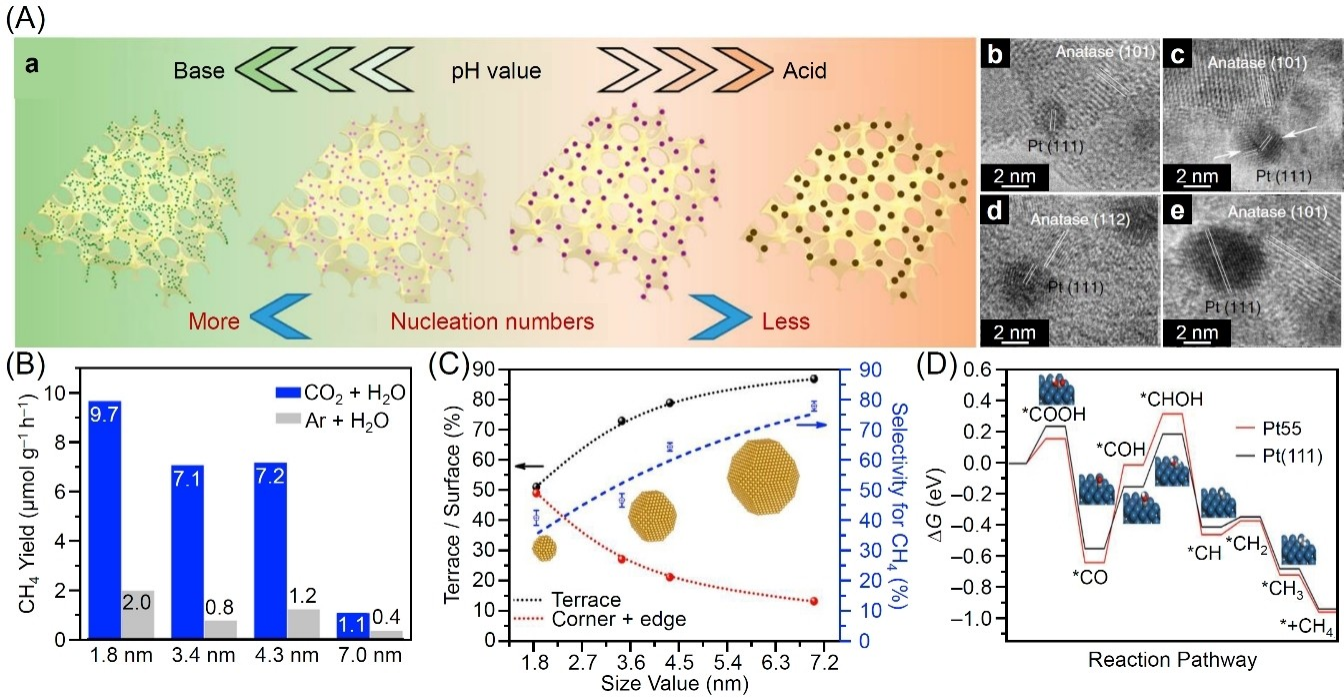
A) Schematic illustration of the acid‐base‐mediated alcohol reduction method for controlling the size of PtNPs (a) and HRTEM images of PtNPs of sizes 1.8 (b), 3.4 (c), 4.3 (d), or 7.0 nm (e) loaded on HTSO. B) CH_4_ yields obtained during CRR on PtNPs/HTSO with different sized PtNPs under different atmospheres. C) Correlations between the selectivity for CH_4_ and surface site proportion as a function of the size of PtNPs. D) Free energy diagrams for the reduction of CO_2_ to CH_4_ using the thermochemical model on Pt(111) surface and Pt55. Reproduced with permission from ref. [38b]. Copyright: 2018. Springer Nature Limited.

Likewise, Zhang and colleagues have reported the importance of controlling the particle size of cocatalysts in improving the catalytic activity of CRR in 2018.^[38b]^ They synthesized PtNPs on porous TiO_2_‐SiO_2_ (HTSO) by a simple acid–base‐mediated alcohol reduction method (Figure 8A). They investigated the relation between the size of the PtNPs and CRR activity on the obtained photocatalysts, and revealed that the photocatalysts loaded with smaller PtNPs (i. e., 1.8 nm) showed higher CH_4_ evolution rate and HER rate than those loaded with larger PtNPs (3.4–7.0 nm; Figure 8B). To rationalize the results obtained, femtosecond transient absorption (fs‐TA) spectroscopy and transient photocurrent response measurements were performed. The results revealed that decreasing the particle size enhanced charge transfer at the metal‐support interface. Specifically, the authors suggested that the Fermi level shifted to the positive side as the particle size became smaller, which facilitated charge transfer at the interface. In contrast, larger PtNPs showed the high selectivity of CH_4_ evolution. When the size of PtNPs was larger, the ratio of terrace sites, such as Pt(111) planes, increased (Figure 8C). Density functional theory (DFT) calculations indicated that the rate‐determining step of *CO to *COH hydrogenation was more likely to occur on such Pt(111) surfaces (Figure 8D), thus explaining the higher CH_4_ selectivity displayed by the larger PtNPs. These results indicate that changes in the exposed crystal faces associated with size changes of the cocatalyst also have a significant effect on the selectivity of CRR. The study demonstrates that 1) the smaller particle size of the PtNPs improves the catalytic activity of CRR and HER but 2) the generation of terrace sites is required on the surface of the cocatalyst for improving the selectivity of CH_4_ evolution.

### 4.2 Chemical composition control

Alloying of the cocatalyst improves the adsorption of intermediates and facilitates the progression of multi‐electron reactions, resulting in the improvement of the catalytic activity and selectivity in CRR.[^38c^, ^39^]

**Figure 9:**
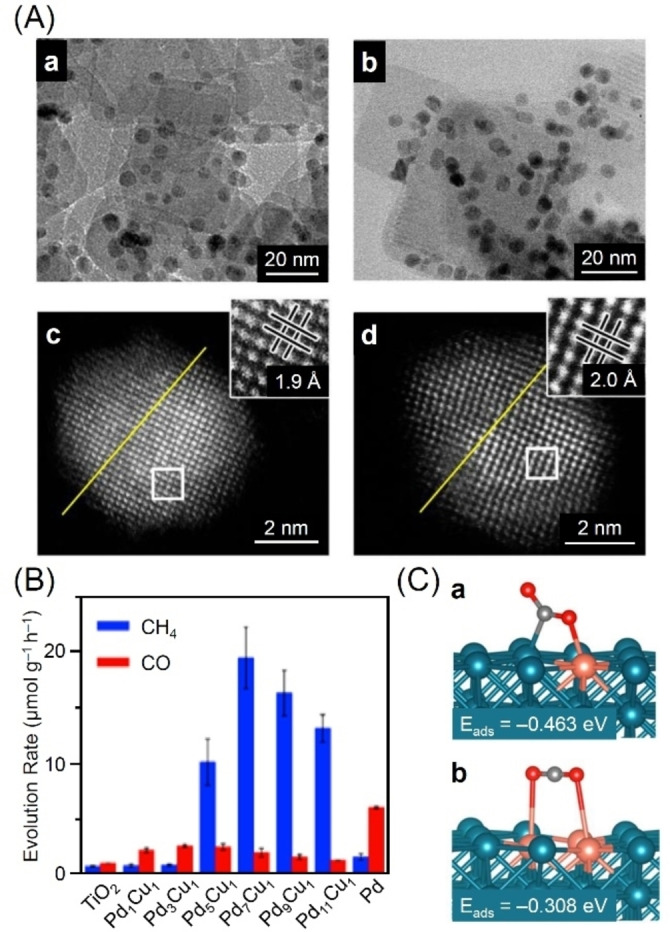
A) TEM images of Pd_1_Cu_1_ NPs/TiO_2_ (a) and Pd_7_Cu_1_ NPs/TiO_2_ (b) and HAADF‐STEM images of Pd_1_Cu_1_ NPs (c) and Pd_7_Cu_1_ NPs (d). The insets in (c) and (d) show atomic‐resolution images taken from the regions indicated by the boxes. B) Average evolution rates of CH_4_ and CO during CRR on different photocatalysts in the presence of H_2_O. C) Most favorable configurations and adsorption energies of CO_2_ at an isolated Cu atom (a) and two neighboring Cu atoms (b). Cu: orange, Pd: blue, C: gray, and O: red. Reproduced with permission from ref. [39a]. Copyright: 2017, American Chemical Society.

Xiong and colleagues successfully obtained alloy cocatalysts with isolated Cu atoms within PdNPs on TiO_2_ in 2017.^[39a]^ Furthermore, the authors found that the resulting PdCu alloy NPs‐loaded photocatalysts (Pd_*x*_Cu_1_ NPs/TiO_2_; *x*=1, 3, 5, 7, 9, or 11) exhibited higher CH_4_ selectivity than PdNPs/TiO_2_ photocatalyst in which pure PdNPs were loaded as cocatalysts. The Pd_*x*_Cu_1_ NPs/TiO_2_ (*x*=1, 3, 5, 7, 9, or 11) photocatalysts with different alloy ratios were obtained by controlling the concentrations of the Pd and Cu salt precursors. Transmission electron microscopy (TEM) images (Figure 9Aa, b) and high‐angle annular dark‐field scanning TEM (HAADF‐STEM) images (Figure 9Ac, d) showed that Pd_*x*_Cu_1_ alloy cocatalysts were loaded on TiO_2_ with similar shape and size (average particle size: ∼6 nm) regardless of the Cu content. Among the Pd_*x*_Cu_1_ NPs/TiO_2_ photocatalysts examined, Pd_7_Cu_1_ NPs/TiO_2_ displayed the highest CH_4_ evolution rate (19.6 μmol g^−1^ h^−1^) and selectivity (95.9 %; Figure 9B). Diffuse reflection infrared Fourier transform spectroscopy (DRIFTS) showed that the evolution of intermediates during CRR was enhanced on the Pd_7_Cu_1_ NPs/TiO_2_ surface relative to the Pd_1_Cu_1_ NPs/TiO_2_ surface. First‐principles simulations also showed that CO_2_ was more strongly adsorbed on the cocatalyst surface of the Pd−Cu pair when Cu atoms were surrounded by a higher number of Pd atoms (Figure 9C). These two factors were interpreted as the origin of the increase in the CH_4_ evolution rate and selectivity of the final product when the Cu atoms were isolated in the Pd lattice to form an alloy cocatalyst.

**Figure 10:**
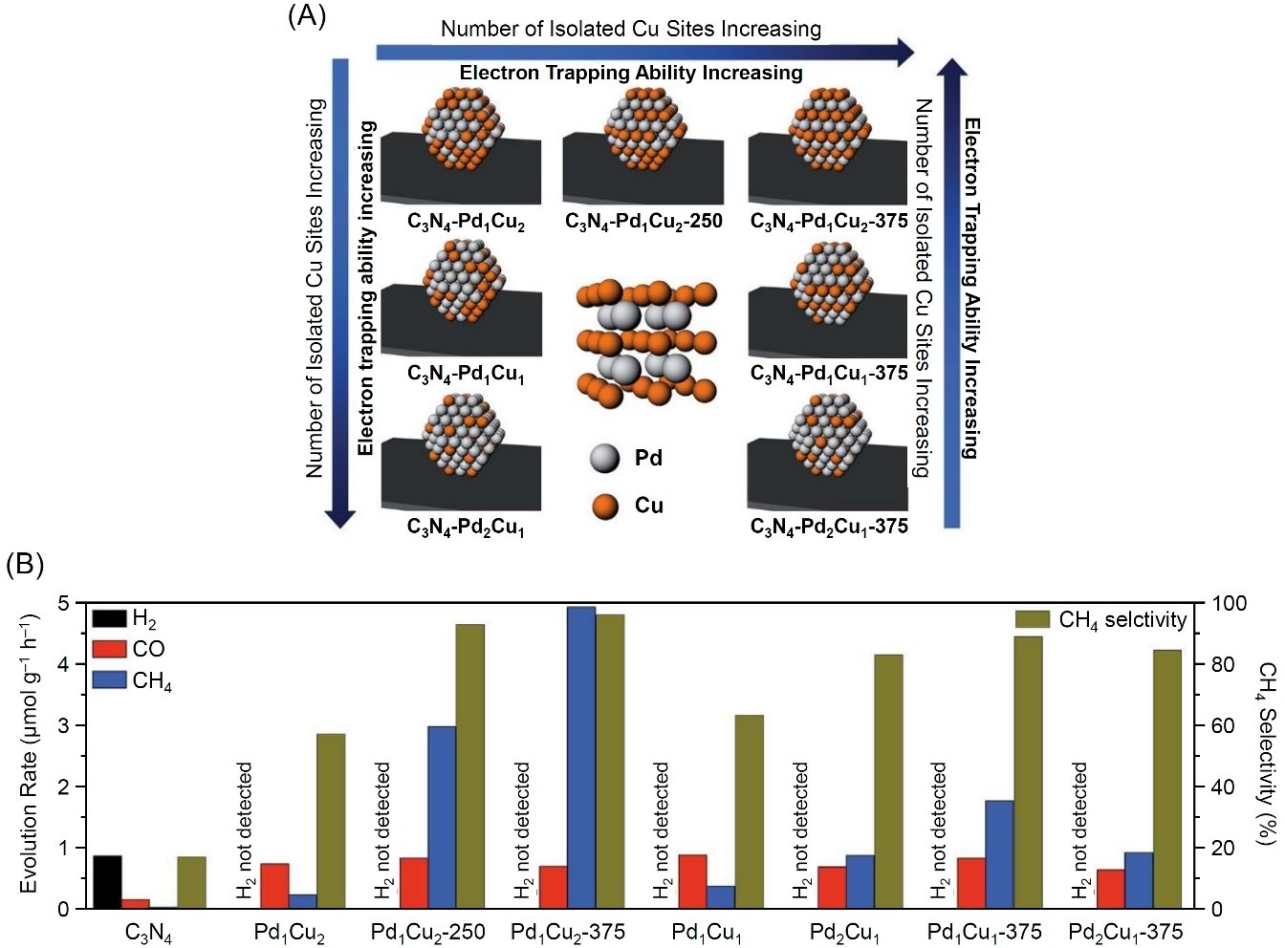
A) Schematic illustrating enhanced electron‐trapping ability and enhanced number of the isolated Cu sites in ordered PdCu cocatalysts prepared at various Pd/Cu molar ratios and at various annealing temperatures; inset: atomic model of the ordered PdCu intermetallic structure. B) Average evolution rates of H_2_, CO, and CH_4_ and CH_4_ selectivity during CRR on g‐C_3_N_4_‐based photocatalysts under visible light irradiation. Reproduced with permission from ref. [39b]. Copyright: 2018, The Royal Society of Chemistry.

Bai and colleagues also reported a study on PdCu alloy cocatalysts with similar metal species in 2018.^[39b]^ The authors investigated the CRR activity of PdCu‐ordered alloy cocatalysts (Figure 10A) formed on g‐C_3_N_4_ nanosheets. The results demonstrated that the Pd_1_Cu_2_ NPs/g‐C_3_N_4_ photocatalyst annealed at 375 °C in H_2_ atmosphere 1) had ordered alloy layers with a body‐centered cubic (bcc) structure (Figure 10A) and 2) had a higher CH_4_ evolution rate (4.95 μmol h^−1^ g^−1^) and selectivity (96.5 %) than the other examined PdCuNPs/g‐C_3_N_4_ photocatalysts with different composition ratios (Figure 10B). The authors further found that in the PdCu‐ordered alloy cocatalyst, 1) the electronic interaction between Pd and Cu was enhanced, thus increasing their electron trapping capacity and 2) isolated Cu sites were exposed on the cocatalyst surface (Figure 10A). The origin of the high catalytic activity and selectivity of the Pd_1_Cu_2_ NPs/g‐C_3_N_4_ photocatalyst was attributed to these two phenomena.

**Figure 11:**
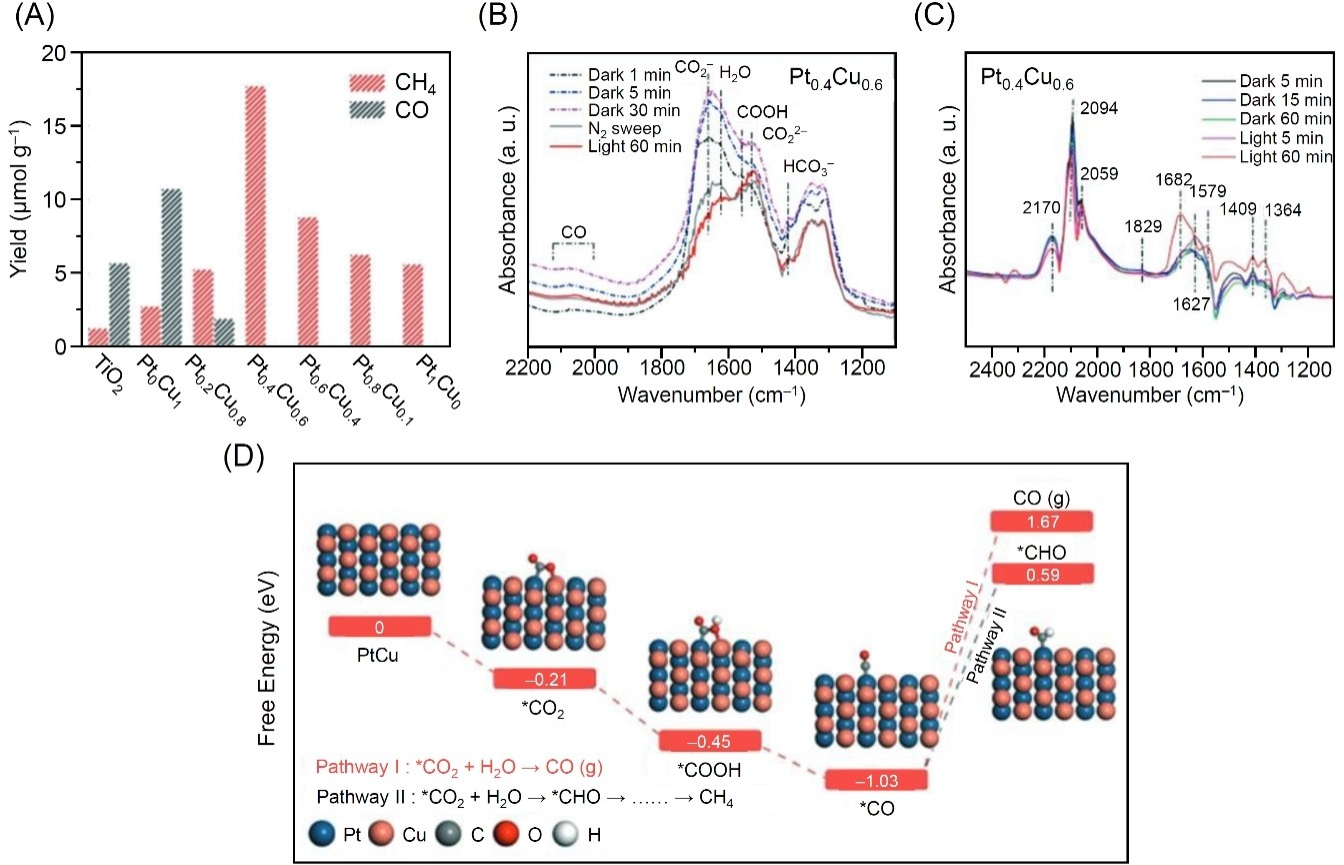
A) CO and CH_4_ yields during CRR over Pt_*x*_Cu_*y*_ NPs/TiO_2_. B) CO_2_ in‐situ FTIR spectra of Pt_0.4_Cu_0.6_/TiO_2_. C) CO in‐situ FTIR spectra of Pt_0.4_Cu_0.6_ NPs/TiO_2_. D) Free energy diagrams of CO_2_ reduction on PtCuNPs. An asterisk denotes the adsorbed intermediate on the substrate. Reproduced with permission from Ref. [39c]. Copyright: 2022, The Royal Society of Chemistry.

In a study by Zhao and colleagues in 2022, the authors investigated the selectivity and mechanism of CRR using photocatalysts loaded with PtCu alloy NPs composed of Pt and Cu.^[39c]^ PtCuNPs/TiO_2_ were prepared by the H_2_ reduction method. X‐ray photoelectron spectroscopy measurements, STEM measurements, and energy‐dispersive X‐ray spectroscopy measurements confirmed the loading of the alloy NPs, consisting of Pt and Cu, on TiO_2_. The results of the catalytic activity study revealed that Pt_0.4_Cu_0.6_ NPs/TiO_2_ did not produce H_2_, and CH_4_ was obtained at 100 % selectivity (Figure 11A). Multiple experiments showed that alloying 1) suppressed the recombination of the photogenerated carriers and 2) enhanced charge transfer from the photocatalyst to the cocatalyst. In‐situ Fourier transform infrared spectroscopy (FTIR) and DFT calculations were performed to elucidate the mechanism of the selective evolution of CH_4_. The results suggested that the selective reduction to CH_4_ occurred by the following mechanism: 1) CO_2_ molecules adsorb chemically on the surface of the catalyst and they trap e^−^, producing the intermediate CO_2_^−^ (Figure 11B); 2) CO_2_^−^ causes the evolution of *CO, an important intermediate species for the generation of CH_4_ and 3) activation and hydrogenation of *CO is promoted (Figure 11C). The authors suggested that in this mechanism, desorption of *CO from the PtCu alloy catalyst surface is difficult because *CO and PtCuNPs form a strong bond (Figure 11D), resulting in increased CH_4_ selectivity and reaction activity.

### 4.3 Morphological control

The shape and crystal facets of the metal cocatalyst have significant effects on adsorption of CO_2_ and thereby the catalytic activity. Therefore, it is important to form cocatalysts with appropriate facets to obtain the desired outcome. Such optimum shape and crystal facets depend on the metal species of the cocatalyst.[^39d^, ^40^]

**Figure 12:**
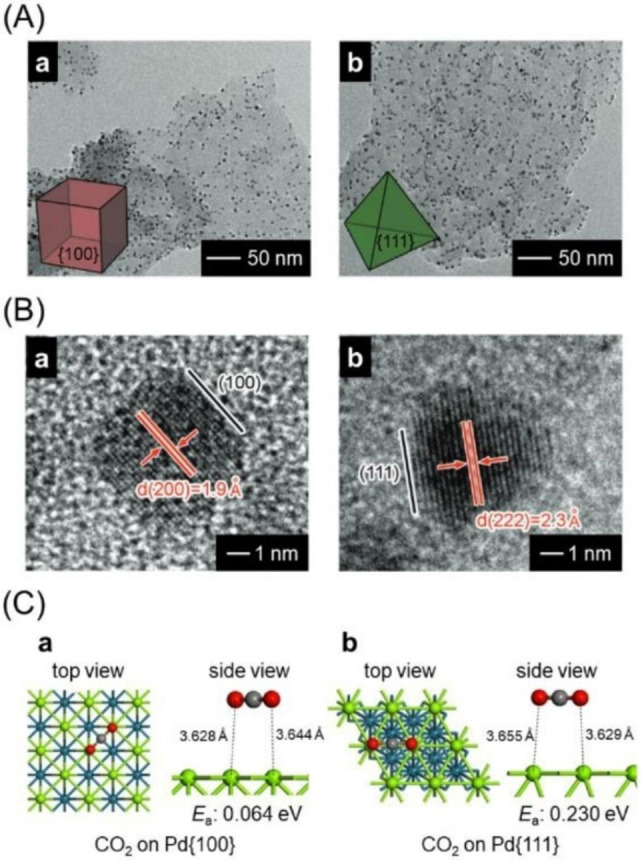
A) TEM and B) HRTEM images of cube‐PdNPs (a) and tetra‐PdNPs (b). C) Optimized configurations of CO_2_ adsorbed onto Pd(100) (a) or Pd(111) (b) facets; the adsorption energy *E_a_* is also noted. Pd: green and blue, C: gray, and O: red. Reproduced with permission from ref. [40a]. Copyright: 2014, The Royal Society of Chemistry.

In 2014, Xiong and colleagues examined two types of photocatalysts loaded with differently shaped PdNP (∼4–6 nm) cocatalysts (cubic; cube‐PdNPs and tetrahedral; tetra‐PdNPs; Figure 12A) to investigate the effect of metal cocatalyst shape on CRR activity.^[40a]^ The PdNPs cocatalysts of different shapes were formed on g‐C_3_N_4_ using appropriate capping agents in the liquid phase. As confirmed from the high‐resolution (HR) TEM images in Figure 12B, the NPs in the form of cube‐PdNPs and tetra‐PdNPs were loaded on the photocatalyst (cube‐PdNPs/g‐C_3_N_4_ and tetra‐PdNPs/g‐C_3_N_4_) and were composed of Pd(100) and Pd(111) planes, respectively. Measurements of the CRR activity revealed that tetra‐PdNPs/g‐C_3_N_4_ displayed approximately four times higher carbon product (CH_4_, C_2_H_5_OH, CO) selectivity than cube‐PdNPs/g‐C_3_N_4_. The photocurrent and photoluminescence measurements indicated that differences in the cocatalyst shape had no effect on charge transfer from g‐C_3_N_4_ to PdNPs. Therefore, simulation calculations were performed, which showed that the Pd(111) facet displayed a higher CO_2_ adsorption energy (*E*_a_; Figure 12C) and a lower activation energy barrier than the Pd(100) facet. These results suggest that loading of tetra‐PdNPs composed of Pd(111) planes is effective in engineering appropriate photocatalysts for CRR using Pd as a cocatalyst element.

**Figure 13:**
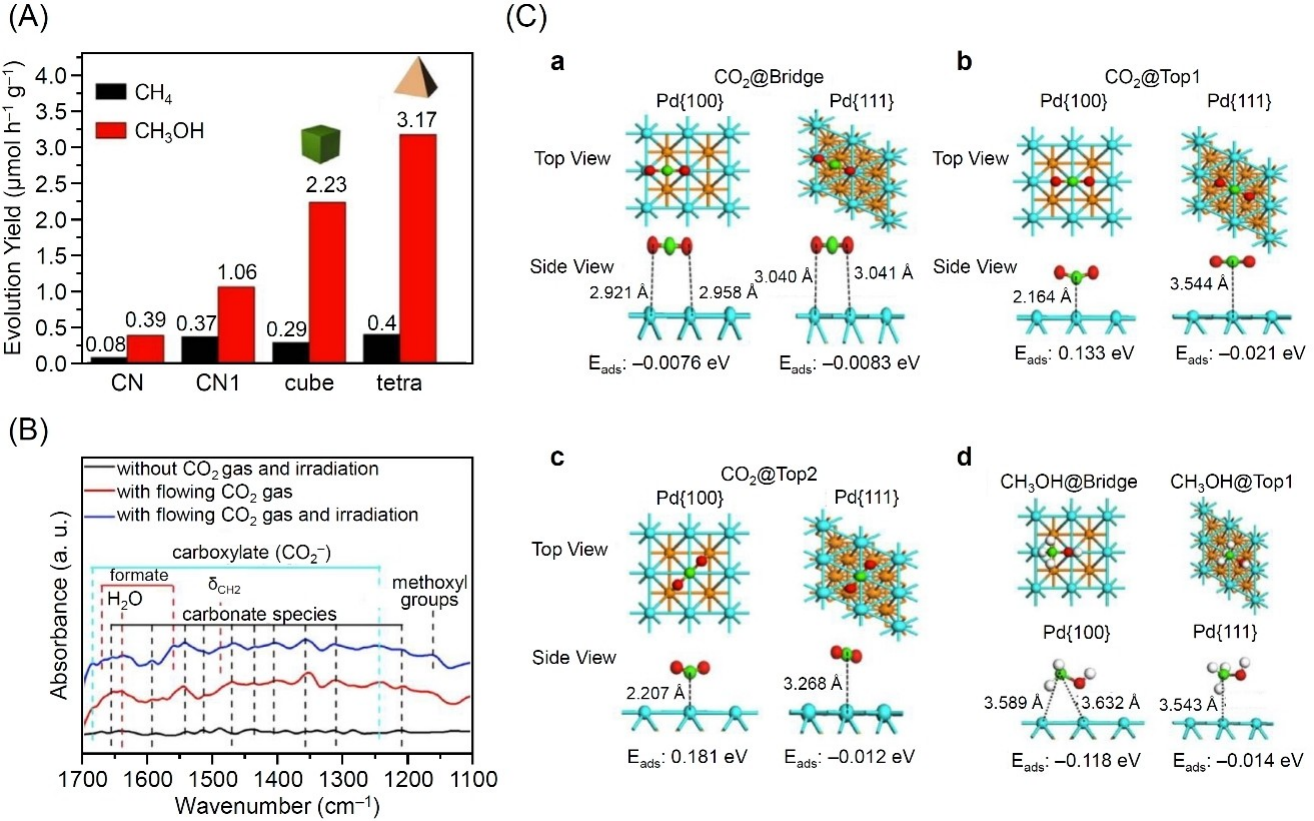
A) Evolution yields of CH_4_ and CH_3_OH during CRR on different photocatalysts – CN: g‐C_3_N_4_ only, CN‐1: photocatalyst subjected to the same treatment but without PdNPs, cube: cube‐PdNPs/g‐C_3_N_4_, and tetra: tetra‐PdNPs/g‐C_3_N_4_. B) In‐situ FTIR spectra of tetra‐PdNPs/g‐C_3_N_4_ subjected to different CRR conditions. C) Optimized geometrical structures of CO_2_ adsorption on Pd(100) facets or Pd(111) facets at the bridge position (a), Top1 position (b), and Top2 position (c), and optimized geometrical structures oof CH_3_OH adsorption on Pd(100) facets or Pd(111) facets (d). Pd: blue and orange, C: green, O: red, and H: white. Reproduced with permission from ref. [40b]. Copyright: 2017, Elsevier B.V.

The effect of cocatalyst shape on selectivity during CRR was also reported by Yu and colleagues in 2017.^[40b]^ PdNPs of different shapes were synthesized using different capping agents and subsequently loaded onto g‐C_3_N_4_ by electrostatic interaction. From the CRR activity measurements, it was found that tetra‐PdNPs/g‐C_3_N_4_ displayed 1.42 times higher CH_3_OH evolution rate than cube‐PdNPs/g‐C_3_N_4_ (Figure 13A). In situ FTIR spectrum of tetra‐PdNPs/g‐C_3_N_4_ during photoirradiation displayed absorption bands related to the evolution of intermediates (HCOOH and HCHO; Figure 13B). This result suggests that CRR on tetra‐PdNPs/g‐C_3_N_4_ surfaces proceeds according to a multistep mechanism involving the evolution of intermediates, such as HCOOH and HCHO, which are subsequently converted into CH_4_ and CH_3_OH. In addition, DFT calculations showed that 1) CO_2_ was more strongly adsorbed on tetra‐PdNPs than on cube‐PdNPs at all sites of the bridge, Top1, and Top2 positions (Figure 13Ca–c) and 2) the product, CH_3_OH, more readily desorbed from the Pd(111) surface than from the Pd(100) surface (Figure 13Cd). They concluded that tetra‐PdNPs/g‐C_3_N_4_ with a Pd(111) facet exhibit high CRR activity due to these factors.

**Figure 14:**
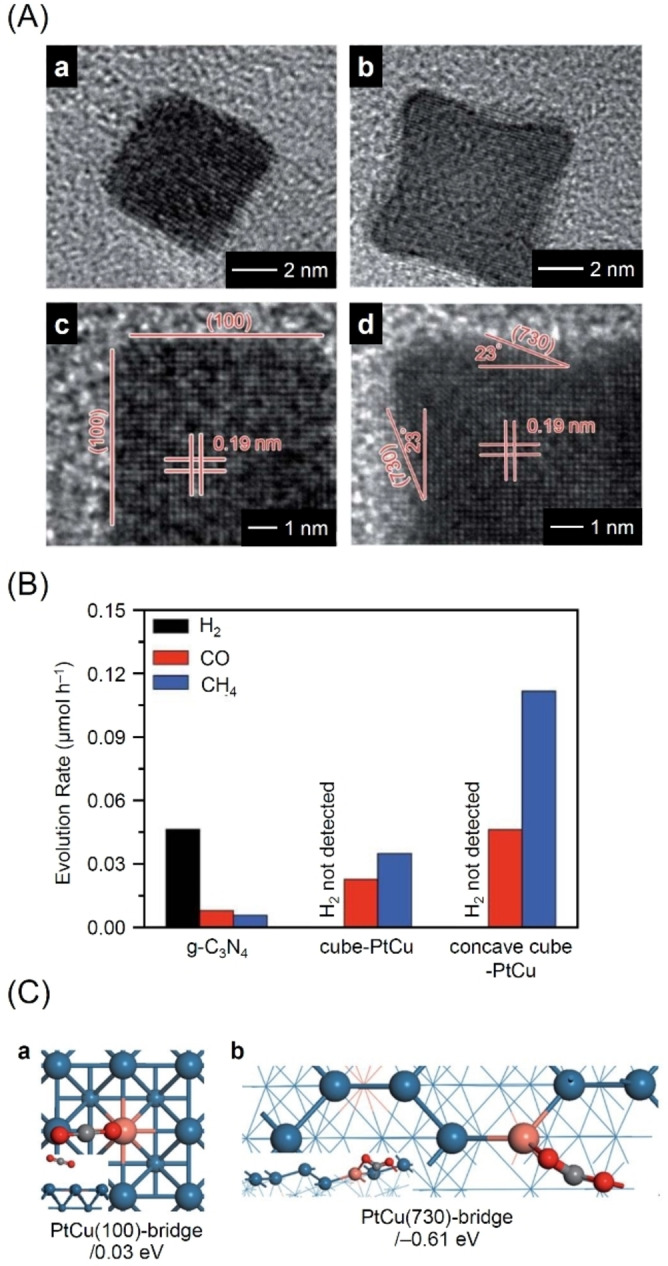
A) TEM (top) and HRTEM (bottom) images of cube‐PtCu (left) and concave cube‐PtCu (right) NPs. B) Photocatalytic H_2_, CO, and CH_4_ evolution rates during CRR on photocatalysts g‐C_3_N_4_, cube‐PtCuNPs/g‐C_3_N_4_, or concave cube‐PtCuNPs/g‐C_3_N_4_. C) Most stable configurations of CO_2_ adsorbed on PtCu(100) or PtCu(730) facets and associated adsorption energies. Pd: blue, Cu: pink, C: gray, and O: red. Reproduced with permission from ref. [39d]. Copyright: 2017, The Royal Society of Chemistry.

This shape dependence was also observed for photocatalysts featuring alloy cocatalysts. In 2017, Bai and colleagues reported that the rate and selectivity of CH_4_ evolution was dependent on the crystal facet of PtCu alloy cocatalysts.^[39d]^ PtCu alloy cocatalysts were formed on g‐C_3_N_4_ by hydrothermal reduction of a solution containing the metal salts (K_2_PtCl_4_, CuCl_2_) and polyvinylpyrrolidone. The shape of the cocatalyst was controlled by adjusting the amount of hydrogen chloride (HCl), which acts as an oxidative etching agent, to obtain cube‐PtCuNPs/g‐C_3_N_4_ (Figure 14Aa) and concave cube‐PtCuNPs/g‐C_3_N_4_ (with a concave surface; Figure 14Ab). The HRTEM measurements revealed that the (100) and (730) facets were exposed in each cocatalyst (Figure 14Ac, d). The concave cube‐PtCuNPs/g‐C_3_N_4_ displayed CO and CH_4_ evolution rates of 0.046 and 0.112 μmol h^−1^, respectively. These evolution rates were respectively about two and three times higher than the CO and CH_4_ evolution rates obtained on cube‐PtCuNPs/g‐C_3_N_4_ (Figure 14B). Furthermore, concave cube‐PtCuNPs/g‐C_3_N_4_ exhibited a CH_4_ selectivity of 90.6 %, which was higher than that (85.9 %) displayed by cube‐PtCuNPs/g‐C_3_N_4_. The DFT calculations suggested that the adsorption energy of CO_2_ was higher on the PtCu(730) facet than on the PtCu(100) facet, indicating that the PtCu(730) facet had superior CO_2_ molecular adsorption capacity (Figure 14C). In addition, there is generally a relatively strong electronic interaction between the Pt atom in the low‐coordinated state and CO_2_. These results explained the high CH_4_ evolution activity and selectivity displayed by concave cube‐PtCuNPs/g‐C_3_N_4_.

### 4.4 Surface structure control

To improve the activity and selectivity of CRR, it is also important to suppress the competing HER and reverse reaction that produces CO_2_. Therefore, several groups have attempted to form cocatalysts with a core–shell structure using two different metal species.^[41]^

**Figure 15:**
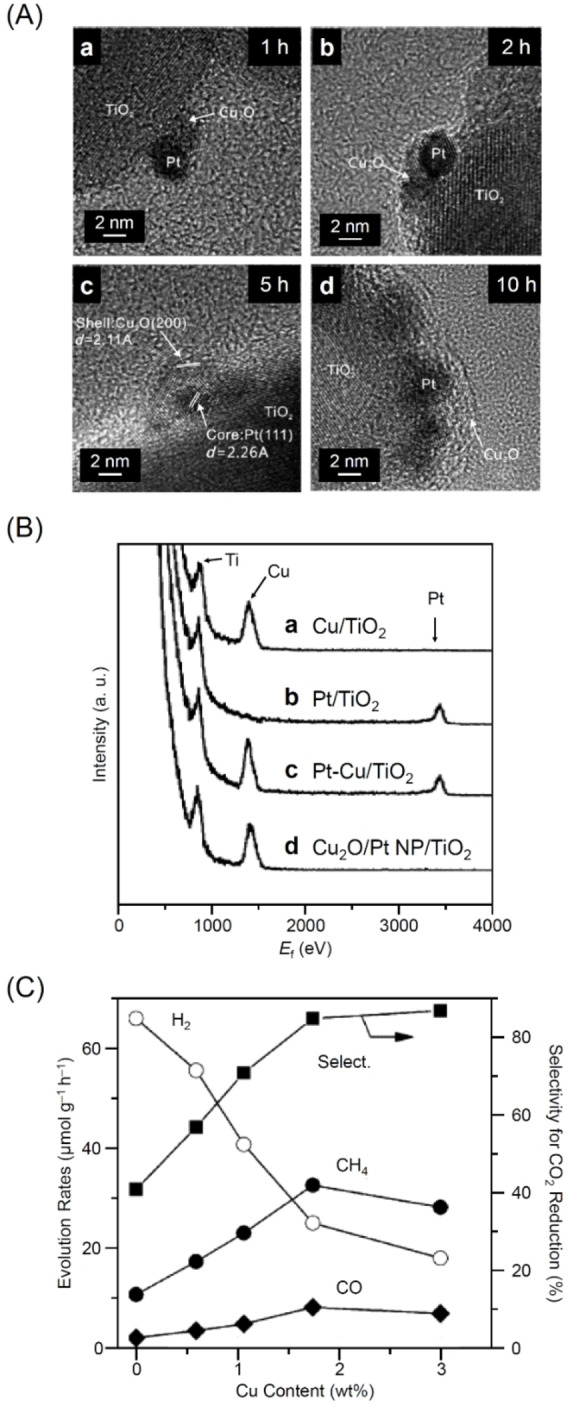
A) HRTEM images of Cu_2_O/PtNPs/TiO_2_‐1 h (a), Cu_2_O/PtNPs/TiO_2_‐2 h (b), Cu_2_O/PtNPs/TiO_2_‐5 h (c), and Cu_2_O/PtNPs/TiO_2_‐10 h (d). B) HS‐LEIS spectra of Cu/TiO_2_ (a), Pt/TiO_2_ (b), Pt−Cu/TiO_2_ (c), and Cu_2_O/PtNPs/TiO_2_‐5 h (d) photocatalysts recorded at 5 keV ^20^Ne^+^. C) Dependence of the photocatalytic behavior on the Cu content in the Cu_2_O/PtNPs/TiO_2_‐5 h catalysts for CRR in the presence of H_2_O. Reproduced with permission from ref. [41a] Copyright: 2013, Wiley‐VCH.

In 2013, Wang and colleagues reported the synthesis of cocatalysts with a core–shell structure composed of Pt and Cu_2_O and their high selectivity for the evolution of CO and CH_4_.^[41a]^ A photodeposition method was used to form PtNPs on TiO_2_ with a particle size of about 3.1 nm. Copper sulfate was then added to form Cu_2_O/PtNPs/TiO_2_, in which PtNPs (core) were covered by Cu_2_O (shell) in a stepwise photodeposition. The thickness of the shell layer composed of Cu_2_O was controlled by the light irradiation time (*x* h) (Cu_2_O/PtNPs/TiO_2_‐*x* h). As confirmed by TEM analysis, the Cu shell completely covered the PtNPs after light irradiation for 5 h (Cu_2_O/PtNPs/TiO_2_‐5 h; Figure 15A). The evolution of the shell layer after 5 h of light irradiation was also confirmed by high‐sensitivity low‐energy ion scattering (HS‐LEIS) measurements (Figure 15B). Cu_2_O/PtNPs/TiO_2_‐5 h, with a Cu content of 3 wt%, showed a CRR selectivity of 85 % (Figure 15C). Within this core–shell configuration, the PtNPs as the core have a high electron capture capacity, while the Cu_2_O as the shell suppresses the reduction of H_2_O to H_2_. Therefore, the core–shell configuration of this Cu_2_O/PtNPs/TiO_2_ photocatalyst appears to be conducive to suppressing the competing HER and enabling CRR on the photocatalyst.

**Figure 16:**
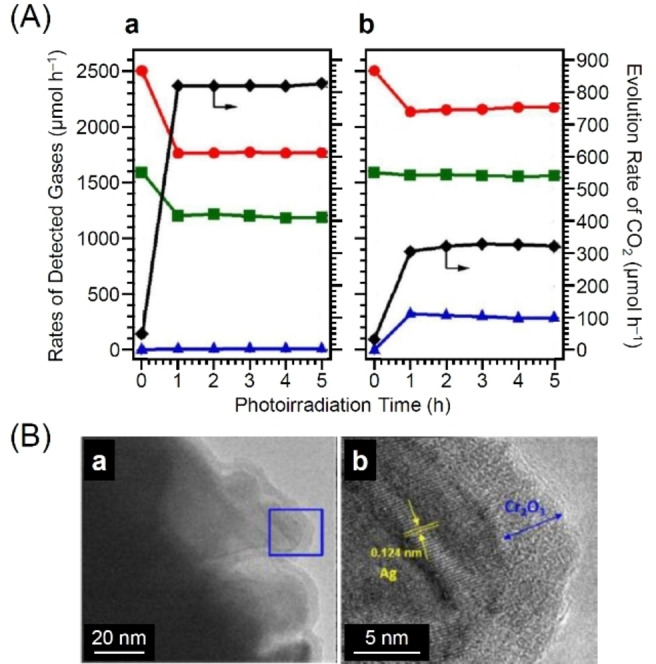
A) Evolution rates of H_2_ (▴), O_2_ (▪), CO (•), and CO_2_ (⧫) of the reverse reaction for the photocatalytic conversion of CO_2_ in H_2_O over AgNPs/Ga_2_O_3_ (a) and Cr(OH)_3_ ⋅ *x*H_2_O/AgNPs/Ga_2_O_3_ (b). Photocatalyst powder: 0.5 g; reaction solution: 1.0 L H_2_O; flowing rates of gases: 20 mL min^−1^ CO/Ar mixture gas (5.0 %), 0.64 mL min^−1^ O_2_, and 9.4 mL min^−1^ Ar; solution: 1.0 L H_2_O; Ag loading amount: 1.0 mol%; Cr loading amount: 1.0 mol%; light source: 400 W high‐pressure Hg lamp. B) TEM (a) and HRTEM (b) images of Cr(OH)_3_ ⋅ *x*H_2_O/AgNPs/Ga_2_O_3_. Reproduced with permission from ref. [41b]. Copyright: 2018, The Royal Society of Chemistry.

In contrast, in the core–shell‐type cocatalyst reported in 2018 by Tanaka and colleagues, the reverse reaction of CRR was significantly suppressed instead of the HER (Figure 16A).^[41b]^ The core–shell‐type cocatalysts consisting of AgNP cores and chromium hydroxide (Cr(OH)_3_) shells were formed on Ga_2_O_3_ by photodeposition (Cr(OH)_3_ ⋅ *x*H_2_O/AgNPs/Ga_2_O_3_). HRTEM analysis confirmed the formation of Cr(OH)_3_ ⋅ *x*H_2_O shell of about 3–5 nm thickness on the AgNP surface (Figure 16B). The obtained Cr(OH)_3_ ⋅ *x*H_2_O/AgNPs/Ga_2_O_3_ photocatalyst exhibited an evolution rate of 480 μmol h^−1^ and a selectivity of 83.8 % for the conversion of CO_2_ into CO. These values were respectively 2.4 and 2.0 times higher than those of AgNPs/Ga_2_O_3_ photocatalyst without a core–shell structure. Cr(OH)_3_ ⋅ *x*H_2_O, which forms the shell layer, is assumed to change to the carbonate compound, Cr_2_(OH)_2*m*_(CO_3_)_(3‐*m*)_ ⋅ *x*H_2_O, in NaHCO_3_ solution. This shell layer of carbonate compounds is believed to provide continuous supply of CO_2_ molecules to the AgNP core, while preventing the approach of O_2_, thereby suppressing the reverse reaction on Cr(OH)_3_ ⋅ *x*H_2_O/AgNPs/Ga_2_O_3_ and leading to high CO selectivity and evolution rate.

**Figure 17:**
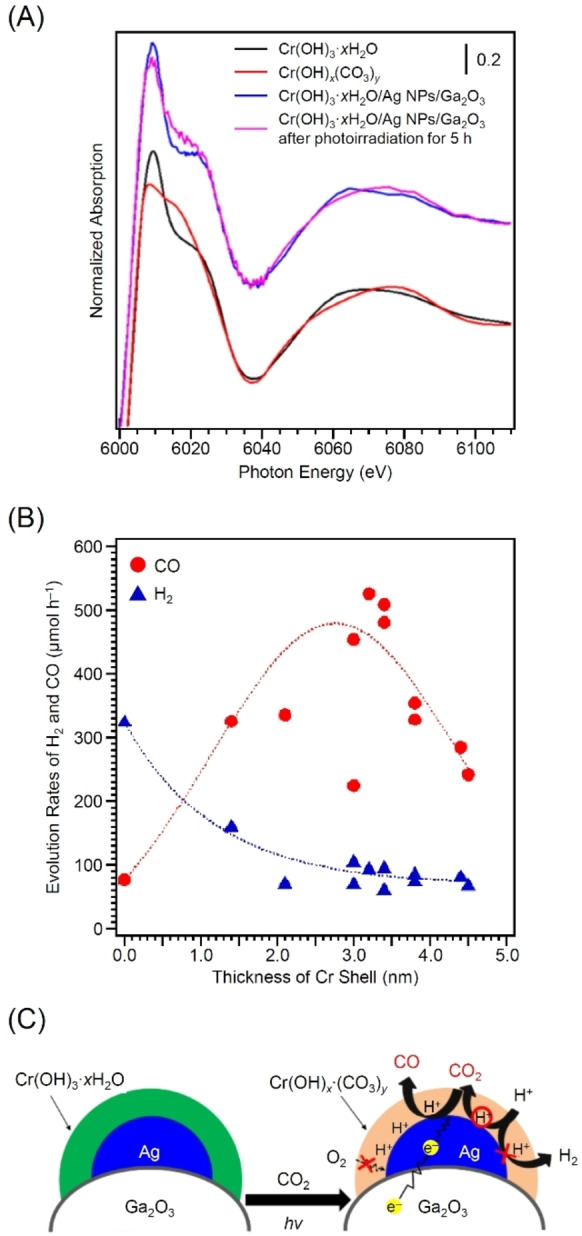
A) Cr K‐edge X‐ray absorption near‐edge structure spectra of Cr(OH)_3_ ⋅ *x*H_2_O (black), Cr(OH)_*x*_(CO_3_)_*y*_ (red), as‐prepared 1.0 mol% Cr(OH)_3_ ⋅ *x*H_2_O/AgNPs/Ga_2_O_3_ (blue), and Cr(OH)_3_ ⋅ *x*H_2_O/AgNPs/Ga_2_O_3_ after photoirradiation for 5 h (pink). B) Dependence of the evolution rates of CO (•) and H_2_ (▴) on the thickness of the Cr(OH)_3_ ⋅ *x*H_2_O shell with various loading amounts of Ag and Cr. Dotted lines represent the data‐fitted curves. C) Schematic illustration of the mechanism of the photocatalytic conversion of CO_2_ into CO on Cr/Ag/Ga_2_O_3_. Reproduced with permission from ref. [41c]. Copyright: 2019, American Chemical Society.

In 2019, Tanaka and colleagues studied the effect of shell thickness on the photocatalytic activity and selectivity for the same photocatalyst.^[41c]^ The studies revealed that 1) high CO evolution rates were obtained when Cr(OH)_3_ ⋅ *x*H_2_O/AgNPs/Ga_2_O_3_ was prepared at Ag : Cr ratios of 1 : 1 and 2) the highest CO evolution rate (525.3 μmol h^−1^) and selectivity (85.2 %) were obtained for photocatalysts loaded with Ag and Cr both at 0.25 wt%. Examination of the chemical composition and shape of the Cr shell on the photocatalyst revealed that Cr(OH)_3_ ⋅ *x*H_2_O was transformed to Cr(OH)_*x*_(CO_3_)_*y*_ during the photocatalytic reaction (Figure 17A). Furthermore, the rate of CO evolution was significantly dependent on the thickness of the shell layer (Figure 17B). When a shell layer with appropriate thickness was formed on the surface of the Ag core cocatalyst, CO_2_ was stably trapped within the active site, allowing CO_2_ to be preferentially reduced to CO (Figure 17C). However, a thicker shell layer would prevent the approach of carbon species and protons to the Ag core. These results reveal that the formation of a shell layer of appropriate thickness is key to achieve high catalytic activity and selectivity. Such functional shells have been reported to also form using carbon[^7b^, ^42^] and NiO.[^41d^, ^41v^]

## 5 Summary and Outlook

The area of CRR with semiconductor photocatalysts is still in its developing stage, with knowledge acquired from extensive studies conducted in the area. To contribute to future advances in engineering CRR photocatalysts with high efficiency, high selectivity, and high durability, this review has provided a summary of the major findings of recent studies conducted in the field, with a focus on the effects of controlling the properties of metal NP cocatalysts on CRR efficiency. The main insights and discussion points are as follows:

1. A decrease in the size of metal NP cocatalysts affords an increase in the number of active sites owing to an increase in the specific surface area. However, if the size is excessively reduced, the electronic states become discretized. Therefore, electron transfer from the photocatalyst to the cocatalyst is disrupted, potentially leading to a reduction in activity. In addition, changes in particle size lead to changes in the exposed crystal facet. Therefore, to achieve high selectivity and activity, the loading of metal NPs of suitable size is important.
2. Alloying PdNPs or PtNPs with Cu suppresses recombination of the photogenerated carriers, enhances charge transfer from the photocatalyst to the cocatalyst, and stabilizes the reaction intermediates, enhancing the selectivity and rate of CH_4_ evolution. If isolated Cu sites and ordered alloying are achieved, the selectivity of the products can be further enhanced.
3. Control of the exposed crystal facets of the metal NP cocatalysts contributes to the improvement of the activity and selectivity of the CRR. In the PdNP cocatalyst, metal NPs with exposed Pd(111) surfaces are suitable cocatalysts for CRR. In the PtCu alloy cocatalyst, exposure of the PtCu(730) surface leads to highly efficient and selective CH_4_ evolution.
4. Suppression of the competing HER or the reverse reaction improves the activity and selectivity of CRR. In particular, the formation of shell layers can be effectively used to suppress these reactions.

Given the knowledge gained to date, further areas remain to be explored, as summarized below, to guide the development of future CRR photocatalysts.

1. CRR is a multi‐electron reaction that employs several electrons simultaneously, and a longer charge separation lifetime contributes significantly to the activity. It is expected that the carrier lifetimes of various photocatalysts will be elucidated by fluorescence lifetime measurements, TA spectroscopy,^[43]^ and time‐resolved microwave spectroscopy,^[44]^ and that photocatalysts and cocatalysts with high charge separation efficiency will be designed and developed based on the knowledge obtained.[^26ba^, ^45^]
2. Previous studies have demonstrated that the catalytic activity can be enhanced when cocatalysts are loaded on suitable crystal facets for the reduction and oxidation reactions.[^28c^, ^30n^, ^30o^, ^46^] Therefore, it is expected that CRR photocatalysts with improved activity and selectivity will be developed in the future by establishing methods to selectively load cocatalysts that have size, alloy structures, crystal facets, and shell structures suitable for achieving high activity and selectivity on the crystal facets of the photocatalysts where CRR occurs.
3. It is expected that the geometric structure of highly active and selective cocatalysts will be revealed at the atomic level by aberration‐corrected TEM and STEM^[47]^ measurements, thereby providing a clearer understanding of the effect of surface structure on catalytic activity.
4. Atomically precise metal nanoclusters (NCs),^[48]^ whose geometric/electronic structures have been revealed by single‐crystal X‐ray structural analysis and DFT calculations have been reported to be highly active cocatalysts for photocatalytic water‐splitting reaction and other catalytic reactions. In addition, the use of atomically precise metal NCs as cocatalysts is also beneficial to obtain deeper understanding of the reaction mechanisms. Indeed, we have succeeded in revealing the reason why Pd doping of AuNC cocatalysts decreases the water‐splitting activity, whereas Pt doping of AuNC cocatalysts increases it by using the atomically precise metal NCs as a cocatalyst.^[3d]^ Moreover, single‐atom (SA) catalysts have been reported to be efficient cocatalysts for photocatalytic CRR,^[49]^ and their use also seems to be beneficial for obtaining deeper understanding of the reaction mechanisms. In future studies, it is expected that deeper understanding will be obtained of the reaction mechanisms in CRR by using the atomically precise metal NCs and SAs as cocatalysts and through performing operando measurements^[50]^ such as in‐situ X‐ray absorption fine structure, in‐situ hard X‐ray photoelectron spectroscopy, and DRIFTS.
5. DFT calculations are important for understanding the rate‐limiting step of a reaction.^[51]^ However, at present, metal NP cocatalyst structures are often calculated in a simplified manner owing to computational cost issues. In the future, theoretical calculations on actual cocatalysts are expected to be conducted, providing deeper understanding of the reaction mechanisms and thereby providing clear design guidelines for the development of highly active and selective catalysts.
6. It is expected that researchers in the field of photocatalysis, as well as those in the fields of metal NC chemistry,^[52]^ surface spectroscopic chemistry,^[53]^ and theoretical chemistry^[54]^ will actively engage in the development of materials for application in the CRR, thereby significantly advancing the field of CRR photocatalysts.
7. Although the number of published papers on the photocatalytic conversion of CO_2_ increases year by year, the current state of the art is uneven. Namely, of the previous studies, some do not satisfy the requirements of the field of photocatalytic conversion of CO_2_. The important requirements are as follows: 1) isotope experiments should show the carbon source of the reduction product to be CO_2_ (not contamination); 2) the competing H_2_ production must also be analyzed accurately, and the number of excited electrons consumed in the production of the CO_2_ reduction products must be greater than those in H_2_ production; 3) the oxidation product (in most cases, O_2_; the oxidation product of H_2_O) must be analyzed accurately, and the ratio of excited electrons to holes consumed in the reaction must be 1. In future studies, these requirements are expected to be inevitably satisfied to avoid misunderstanding and thereby establish clear design guidelines for high‐performance CRR photocatalysts.

## Conflict of interest

There are no conflicts to declare.

6

## Biographical Information

*Tokuhisa Kawawaki is a Junior Associate Professor in the Department of Applied Chemistry at Tokyo University of Science. He received his Ph. D. degree in applied chemistry from the University of Tokyo in 2015. In 2016, he worked as a Japan Society for the Promotion of Science (JSPS) postdoctoral fellow at the University of Melbourne. He then worked as a JSPS super postdoctoral fellow at Kyoto University. In 2019, he moved to his current University. His current research topics include the synthesis of metal nanoparticles and nanoclusters in solution and their application in photoelectrochemistry and photocatalysis*.

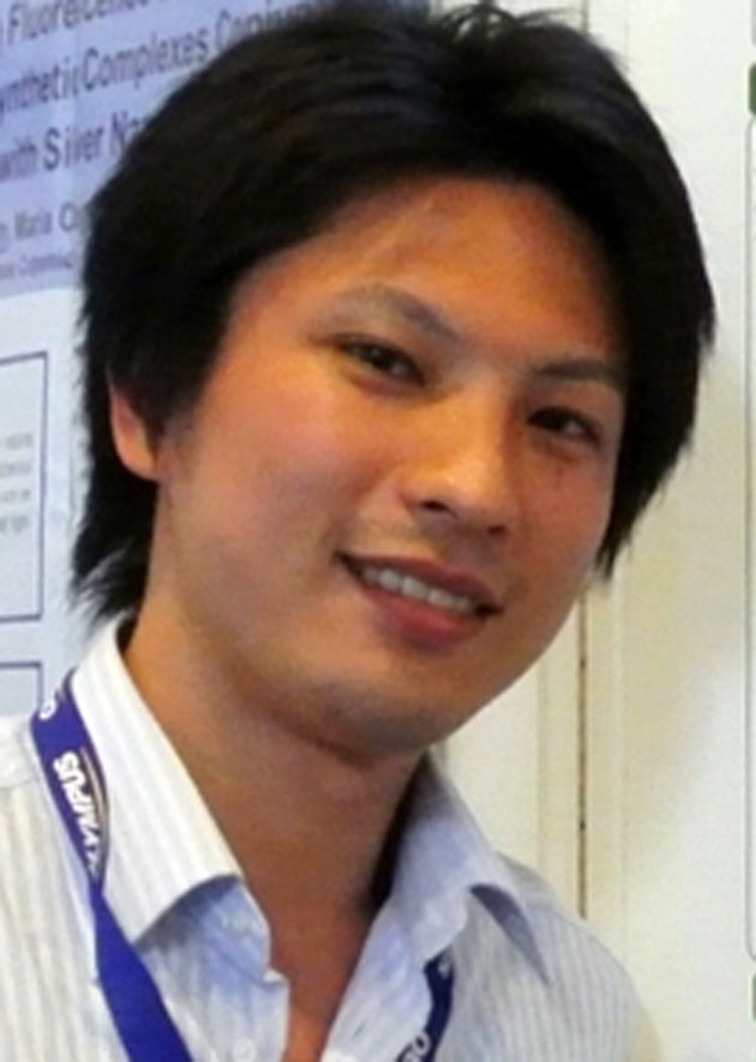

## Biographical Information

*Yuki Akinaga is a masters student in the Negishi group at Tokyo University of Science. He received his B. Sc. degree in chemistry from Tokyo University of Science in 2021. His main research focuses on the development of high‐performance water‐splitting and CRR photocatalysts using metal clusters and single atoms as cocatalysts*.

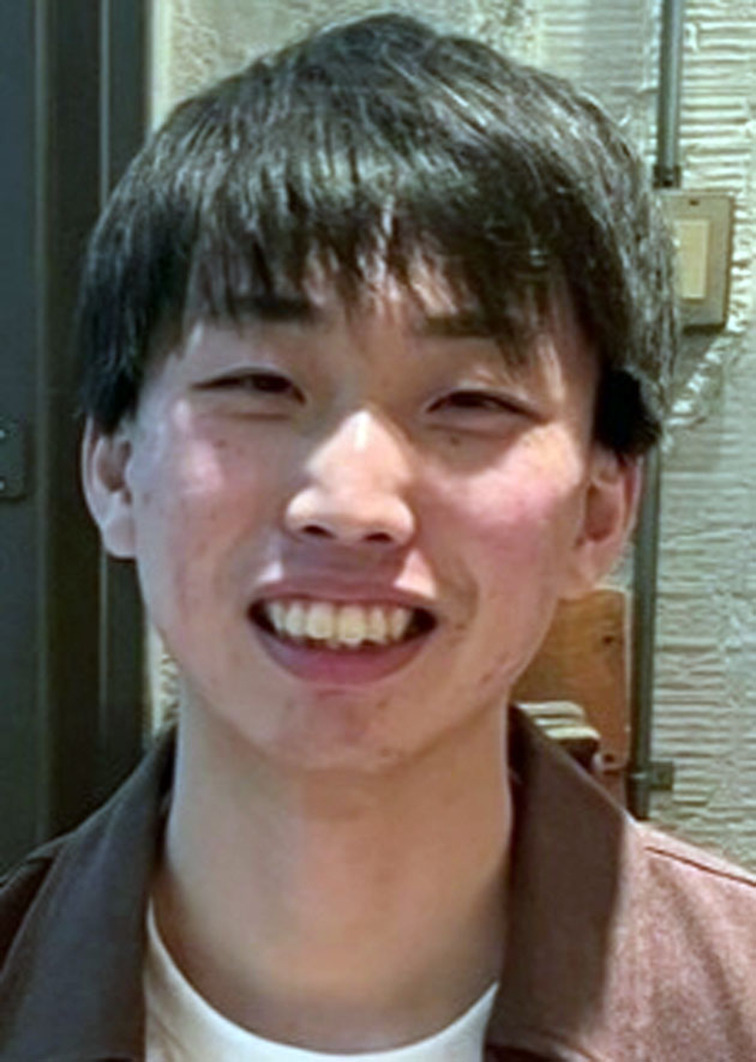

## Biographical Information

*Daichi Yazaki is a masters student in the Negishi group at Tokyo University of Science. He received his B. Sc. degree in chemistry from Tokyo University of Science in 2021. His main research interest is the development of high‐performance water‐splitting photocatalysts using atomically precise metal clusters as cocatalysts*.

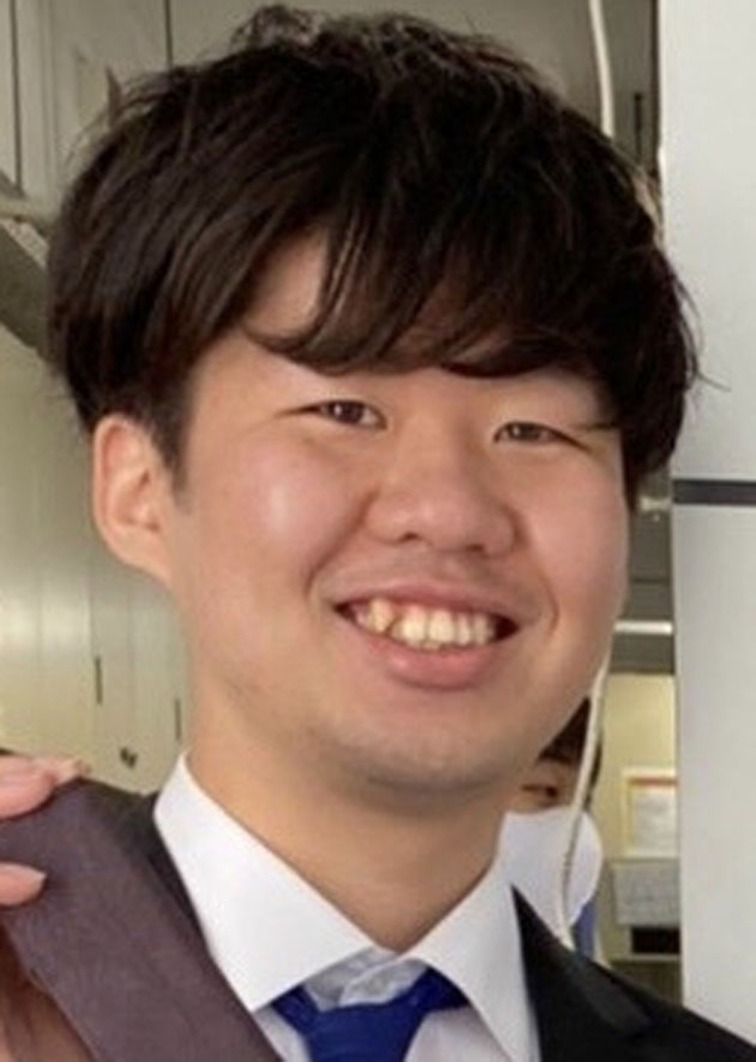

## Biographical Information

*Hinano Kameko is a masters student in the Negishi group at Tokyo University of Science. She received her B. Sc. degree in chemistry from Tokyo University of Science in 2022. Her research focuses on the development of high‐performance water‐splitting and CRR photocatalysts by using metal clusters and single atoms as cocatalysts*.

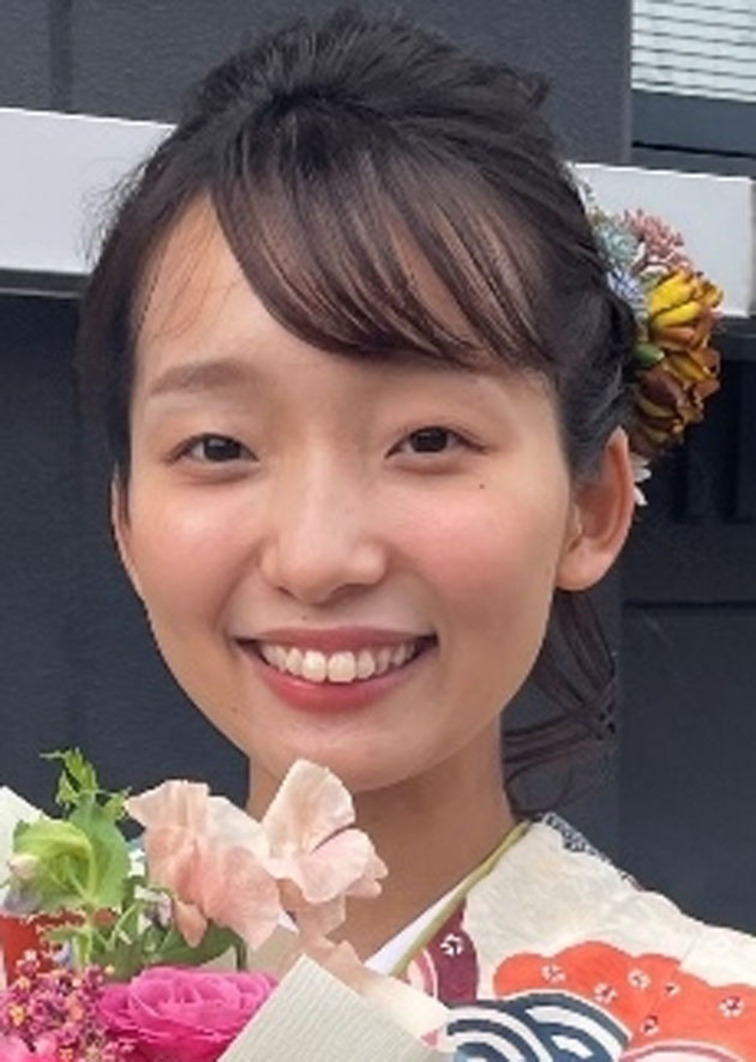

## Biographical Information

*Daisuke Hirayama is a masters student in the Negishi group at Tokyo University of Science. He received his B. Sc. degree in chemistry from Tokyo University of Science in 2022. His main research interest is on the development of high‐performance water‐splitting photocatalysts by using atomically precise metal clusters as cocatalysts*.

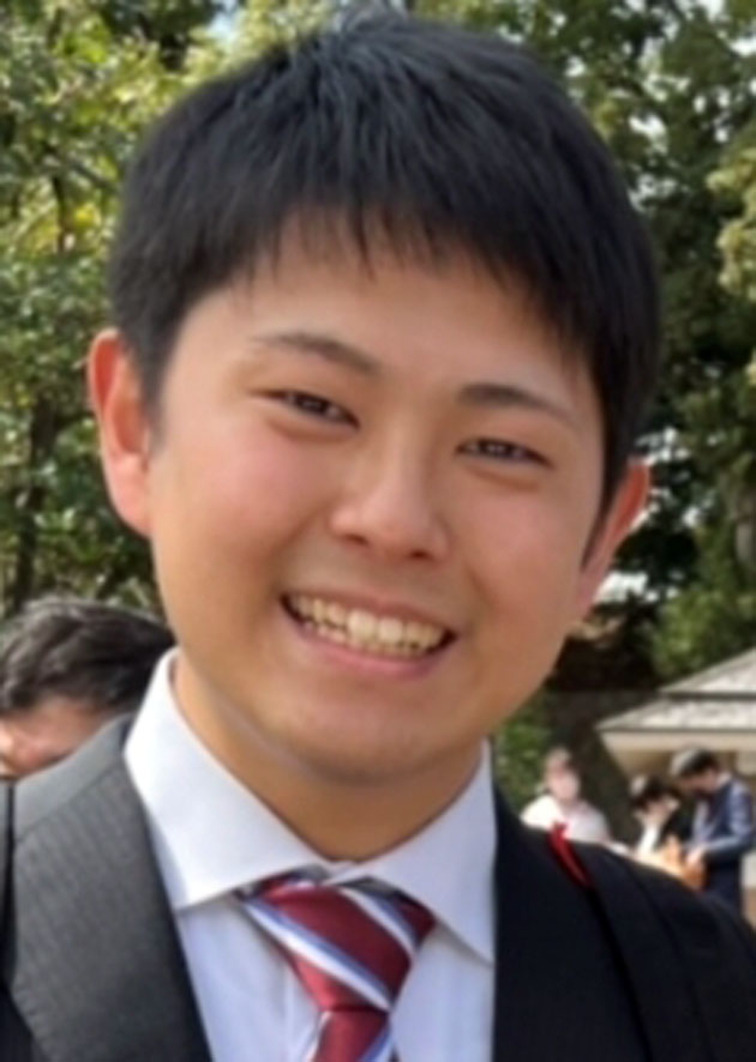

## Biographical Information

*Yuichi Negishi is a professor in the Department of Applied Chemistry at Tokyo University of Science. He received his Ph. D. degree in chemistry in 2001 under the supervision of Prof. Atsushi Nakajima from Keio University. Before joining Tokyo University of Science in 2008, he was employed as an assistant professor at Keio University and at the Institute for Molecular Science. His current research interests include the precise synthesis of stable and functionalized metal nanoclusters for use in energy‐ and environmental‐related application areas*.

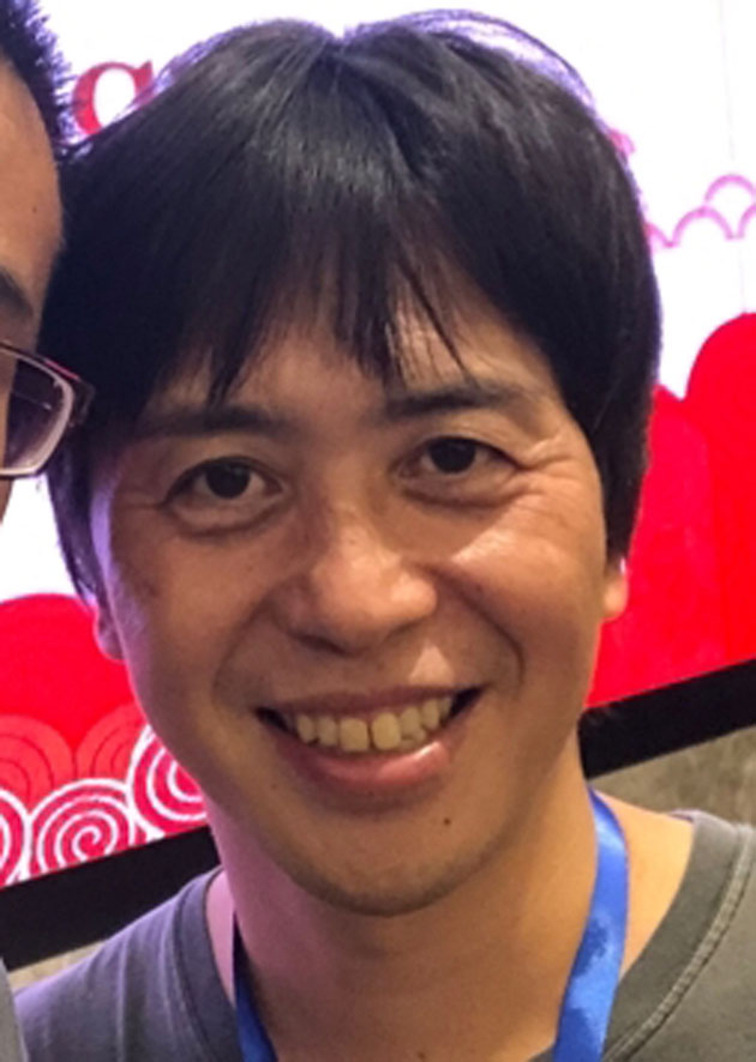

## Data Availability

The data that support the findings of this study are available from the corresponding author upon reasonable request.
